# Hypoxia is a Key Driver of Alternative Splicing in Human Breast Cancer Cells

**DOI:** 10.1038/s41598-017-04333-0

**Published:** 2017-06-22

**Authors:** Jian Han, Jia Li, Jolene Caifeng Ho, Grace Sushin Chia, Hiroyuki Kato, Sudhakar Jha, Henry Yang, Lorenz Poellinger, Kian Leong Lee

**Affiliations:** 1 Cancer Science Institute of Singapore, National University of Singapore, 117599 Singapore, Singapore; 2 Department of Cell and Molecular Biology, Karolinska Institutet, Stockholm, SE-171 77 Sweden

## Abstract

Adaptation to hypoxia, a hallmark feature of many tumors, is an important driver of cancer cell survival, proliferation and the development of resistance to chemotherapy. Hypoxia-induced stabilization of hypoxia-inducible factors (HIFs) leads to transcriptional activation of a network of hypoxia target genes involved in angiogenesis, cell growth, glycolysis, DNA damage repair and apoptosis. Although the transcriptional targets of hypoxia have been characterized, the alternative splicing of transcripts that occurs during hypoxia and the roles they play in oncogenesis are much less understood. To identify and quantify hypoxia-induced alternative splicing events in human cancer cells, we performed whole transcriptome RNA-Seq in breast cancer cells that are known to provide robust transcriptional response to hypoxia. We found 2005 and 1684 alternative splicing events including intron retention, exon skipping and alternative first exon usage that were regulated by acute and chronic hypoxia where intron retention was the most dominant type of hypoxia-induced alternative splicing. Many of these genes are involved in cellular metabolism, transcriptional regulation, actin cytoskeleton organisation, cancer cell proliferation, migration and invasion, suggesting they may modulate or be involved in additional features of tumorigenic development that extend beyond the known functions of canonical full-length transcripts.

## Introduction

Hypoxia is a common feature of tumors that have outgrown their vasculature and constitutes a critical regulatory microenvironment parameter in cancer progression where it drives a number of mechanisms leading to treatment resistance^1–4^. Multiple cellular response pathways are regulated by hypoxia, including angiogenesis, proliferation, metabolism and DNA damage repair^5, 6^. In tumors with hypoxic cores, cancer cells adapt the downstream processes of hypoxia to regulate proliferation, produce ATP, undertake biosynthesis, evade apoptosis and eventually adopt a more aggressive phenotype. The major transcriptional mediators of the downstream hypoxia response are the hypoxia-inducible factors (HIFs), including HIF1α, HIF2α and HIF3α. Under normoxic conditions, the HIFs are hydroxylated by the prolyl hydroxylases (PHDs). This permits the recognition of the hydroxylated proline residues on the HIFs by the von Hippel–Lindau (VHL) tumor suppressor protein, leading to the ubiquitination of the HIFs and subsequent proteasomal degradation^7–9^. Because the hydroxylation of the proline residues by the PHDs depends on the availability of oxygen and 2-oxoglutarate, HIF protein levels are tightly regulated by cellular oxygen levels^10^. Under hypoxic conditions, HIF protein levels increase rapidly due to decreased hydroxylation by the PHDs leading to HIF stabilization. The stabilized HIFs then dimerize with the aryl hydrocarbon receptor nuclear translocator (ARNT) to bind specific hypoxia response elements (HREs) consisting of the core [A/G]CGTG sequence on hypoxia target genes^11^. With the recruitment of the co-activators CREB-binding Protein (CBP) and p300, this leads to the transactivation of HIF target genes^12^. To date, a number of transcriptome analyses have identified many well conserved hypoxia targets such as *VEGFA*, *MXI1*, *PDK1* and *BNIP3*, that play prominent roles in angiogenesis, cell growth, glycolysis and apoptosis in numerous cell types^5, 13–15^.

Nevertheless, in addition to the transcriptional regulation of a vast network of thousands of hypoxia target genes^13^, hypoxia also regulates post-transcriptional modifications, including alternative splicing of pre-mRNA which remains poorly understood. It is estimated that over 95% of human genes undergo alternative splicing, greatly extending the diversity of the human transcriptome and proteome^16^. Different protein isoforms translated from alternatively spliced transcripts may differ in their cellular localization, stability and functions. Alternative splicing can also introduce pre-mature stop codons into transcripts, triggering nonsense-mediated decay (NMD), thereby regulating mRNA and eventually protein expression levels^17^. Aberrant alternative splicing profiles are frequently observed in cancers. Mis-regulated alternative splicing in cancer cells could therefore produce or suppress specific protein isoforms, some of which promote cancer cell survival, proliferation and metastasis. It has been demonstrated that hypoxia may be a potential driver of aberrant alternative splicing in breast cancer carcinogenesis. Hypoxia induces the expression of Cysteine rich 61 (CYR61), a tumorigenic factor that is correlated with breast cancer progression^18^ and shifts Cyr61 alternative splicing towards a functionally active intron exclusion isoform^19^. Furthermore, in triple negative breast cancer cells, hypoxia and HIF1α regulate two splicing variants of CD44, that may contribute to the stem cell-like phenotype of cancer cells^20^. This suggests that as a tumor microenvironmental selective pressure, hypoxia may drive the alternative splicing of many genes to promote oncogenic mechanisms beyond what is known about canonical full-length hypoxia-induced transcripts.

To date, a small number of studies have examined the global hypoxia-regulated alternative splicing landscape using exon arrays in endothelial and liver cancer cell lines as well as in cartilage endplate-derived stem cells. Nine genes involved in angiogenesis-mediated cytoskeletal remodeling (*CASK, ITSN1, LARP6, SPTAN1, TPM1* and *ROBO1*), membrane anchor synthesis (*PIGN*) and gene expression regulation (*CUGBP1* and *MAX*), were identified to be differentially spliced under hypoxia in human endothelial cells^21^. Another genome-wide exon array analysis found that in human hepatoma Hep3B cells, hypoxia regulated the alternative splicing of HIF and non-HIF regulated genes including *CA9, ANGPTL4, PDK1 WNK1, P4HA2, PLOD2* and *ENO2* involved in metabolism, angiogenesis and other processes^22^. Finally, a third study examining the differential gene expression and alternative splicing that occurs during the chondrogenic differentiation of cartilage endplate–derived stem cells in hypoxia also led to the identification of a large number of hypoxia-induced alternative splicing events^23^. *CD44*, *CADM1*, *FGFR3*, *FAM65B*, *TGFBR2*, *RTCA* and *PBLD* were among the splicing targets that may be involved in cartilage development (*FGFR3*) but intriguingly, also in tumorigenic function (*CADM1* and *CD44*). Although there is evidence that hypoxia also promotes oncogenic aberrant splicing in breast cancers^19, 20^, there have been no genome-wide studies of hypoxia-regulated alternative splicing in breast cancer cells that are known to be highly responsive to hypoxia^24, 25^. It has been estimated that over 40% of breast cancers are hypoxic based on the stabilized expression of HIF-1α^26, 27^. The expression of hypoxia markers in breast cancers, such as HIF-1α and CA9, correlates with a more aggressive disease type and poor prognosis^26–29^. The hypoxic microenvironment promotes angiogenesis, cancer cell migration and invasiveness in breast cancer^30–33^, but to what extend these processes are regulated by hypoxia-induced alternative splicing remains unclear. Therefore, to gain a comprehensive understanding of genome-wide hypoxia-regulated alternative splicing and additional insights into the oncogenicity of human breast cancer cells, we performed RNA-Seq on hypoxia treated breast cancer cells and demonstrated that extensive splicing changes occur under hypoxia over and above gene expression changes. We identified 2005 and 1684 significantly regulated alternative splicing events during acute and chronic hypoxia in MCF7 cells. Interestingly, our results suggest that hypoxia predominantly drives intron retention compared to other splicing types. Some of the splicing targets, including *LDHA*, *TNFSF13* and *ARHGAP4* for intron retention, *MARCH7*, *PCBP2* and *LRCH3* for exon skipping and *VGLL4*, *AHNAK* and *NFE2L1* that are subjected to alternative first exon usage may potentially contribute to cancer cell hypoxic adaptation by altering cellular metabolism, transcriptional regulation, actin cytoskeleton organization and promoting cancer cell proliferation, migration and invasion. The identification of these splicing targets provides novel insights into the oncogenic processes driving breast cancer cells and potentially new markers and therapeutic targets in the management of the disease.

## Results

### Hypoxia induces global changes in the gene expression of breast cancer cells

**Figure 1:**
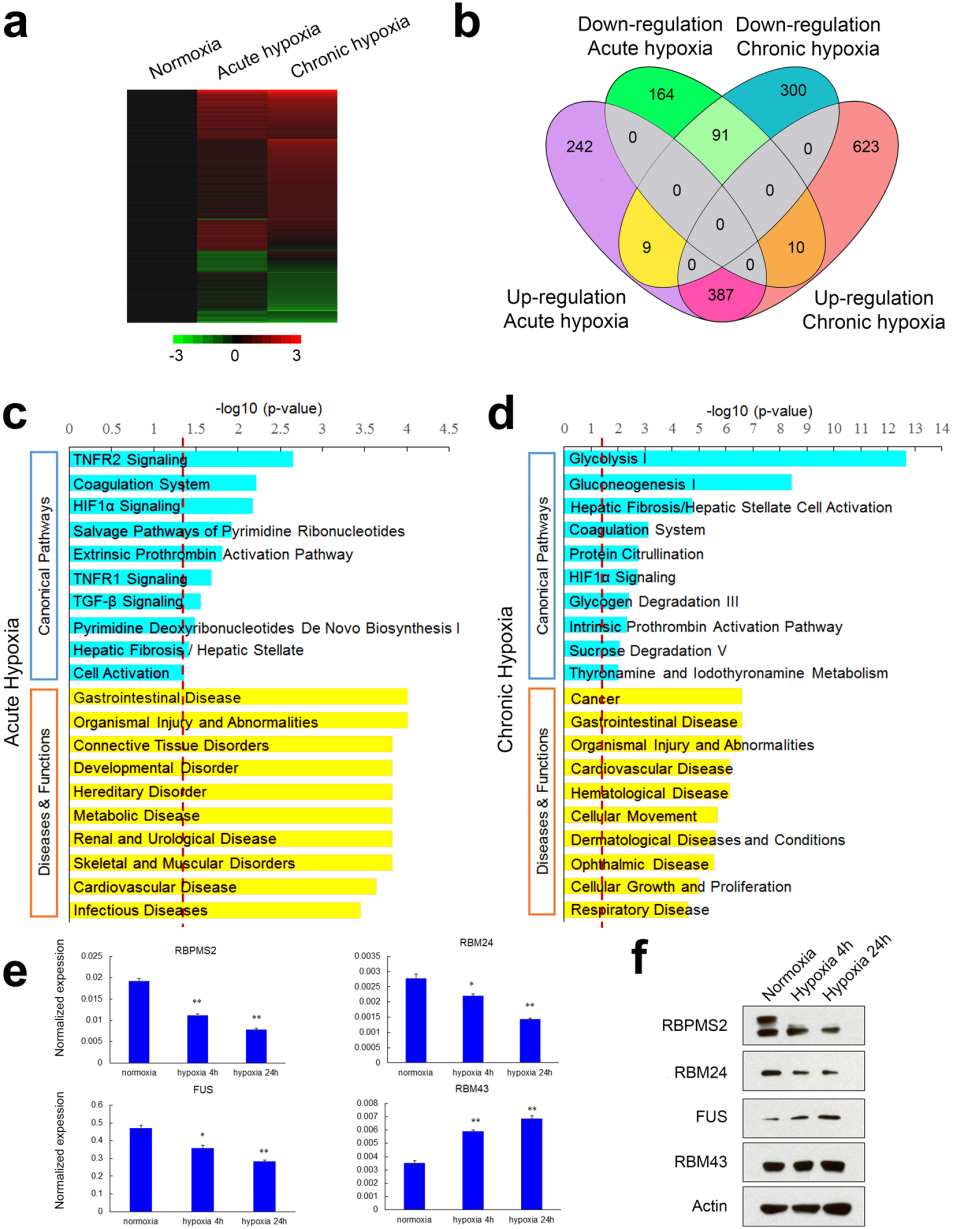
Hypoxia regulates gene expression in MCF7 cells. (**a**) Heat map of target genes identified from RNA-Seq of n = 1 samples that are significantly dysregulated by ≥1.5-fold during acute and chronic hypoxia compared to the normoxia control. Color bar shows fold difference on a Log_2_ scale in red for upregulation and green for downregulation. (**b**) 4-set Venn diagram overlaps of differentially expressed genes (≥1.5-fold) during acute and chronic hypoxia that are up- or downregulated. (**c**) and (**d**) Gene ontology (GO) analysis of hypoxia regulated targets during (**c**) acute and (**d**) chronic hypoxia showing enrichments in canonical pathways as well as diseases and functions as generated by Ingenuity Pathway Analysis (IPA). Red lines indicate p-value of 0.05 for Fisher’s exact test. (**e**) Real-time qPCR analysis of the mRNA expression of the splicing factors *RBPMS2, RBM24, FUS* and *RBM43* in normoxia, acute and chronic hypoxia. The expression of the housekeeping reference gene *PPIA* was used as a control. All qRT-PCR analysis was performed in three biological replicates (n = 3). Error bars indicate SEM. Statistical significance was evaluated with Student’s t-test. * indicates p-value < 0.05, ** indicates p-value < 0.01. (**f**) Western blot analysis of the protein expression of the splicing factors *RBPMS2, RBM24, FUS* and *RBM43* in normoxia, acute and chronic hypoxia. Actin was used as a loading control.

Hypoxia consists of both an acute phase primarily mediated by HIF1α while HIF2α levels increase substantially in the chronic phase^34^. To exclude that any changes in gene expression and alternative splicing could be due to cell death induced by hypoxia, we performed apoptosis assays on the MCF7 cells under normoxia and hypoxic conditions (Supplemental Fig. S2a). Under both acute and chronic hypoxia, less than 2% of the cell populations were found to be in the early and late apoptotic stages and were comparable to the normoxic controls. This suggested that hypoxia did not induce any changes in cell death and therefore this was not a significant phenomenon. Subsequently, we identified the global changes in both gene expression and alternative splicing during hypoxia for the acute and chronic phases. RNA-Seq was carried out on total RNA extracted from MCF7 human breast cancer (ER+, PR+, HER2−) cells cultured in normoxia (21% O_2_, 24 h), acute (1% O_2_, 4 h) and chronic hypoxia (1% O_2_, 24 h) for n = 1 replicate. Both gene expression (Fig. 1e) and alternative splicing (Supplementary Figure S2d) identified from the sequencing results were later validated by real-time qPCR for n = 3 replicates.

Approximately 180 million paired-end 91 base pair reads (>5 times human genome coverage) were sequenced from each sample and mapped to the human genome (hg19) for high sensitivity detection of rare transcripts and accurate quantitation of spliced junction counts with high statistical significance. During acute hypoxia, 638 genes were induced and 265 suppressed by at least 1.5-fold compared to the normoxia control. In chronic hypoxia, 1020 genes were induced and 400 genes were downregulated (Fig. 1a). Therefore, there is a predominance of gene upregulation in both the acute (70.7% of genes differentially expressed by >1.5-fold) and chronic (71.8%) phases of hypoxia in breast cancer cells which is consistent with HIF function as primarily an activator of gene expression^5, 6^. Furthermore, 91 genes were downregulated in both acute and chronic hypoxia and another 387 genes were upregulated. Only 9 upregulated genes in acute hypoxia were downregulated in chronic hypoxia and 10 downregulated genes in acute hypoxia were upregulated in chronic hypoxia (Fig. 1b). 164 and 242 genes were specifically down- or upregulated only during acute hypoxia while another 300 and 623 genes were uniquely down- or upregulated during the chronic phase. This supports that breast cancer cells also undergo different phases of hypoxic induction with substantive differences over time that are consistent with differential HIF1α/HIF2α activities.

As expected, classical hypoxia inducible genes, such as *CA9*, *PDK1*, *ADM* and *BNIP3* ^13–15^, were among the most highly induced genes. Gene ontology analysis of up- and downregulated genes using the Ingenuity Pathway Analysis (IPA) platform showed genes involved in TNFR signaling, coagulation system and HIF1α signaling itself, were significantly enriched in acute hypoxia, while genes involved in glycolysis, gluconeogenesis I and HIF1α signaling were significantly enriched during chronic hypoxia (Fig. 1c and d). In terms of functional and disease processes, we found that genes involved in the IPA classification of organismal injury and abnormalities, including cancer processes, metabolic disease and carbohydrate metabolism were significantly regulated in both acute and chronic hypoxia (Fig. 1c and d).

In addition, the RNA-Seq analysis showed that the expression of 4 genes (*RBPMS2, RBM24, FUS* and *RBM43*) encoding proteins with RNA binding domains of which *FUS* ^35^ and *RBM24* ^36, 37^ have known roles in splicing were also regulated by hypoxia. To validate the RNA-Seq results, qRT-PCR was performed and this confirmed that the expression of *RBM43* was significantly upregulated while the expression of *FUS*, *RBPMS2* and *RBM24* were significantly downregulated during hypoxia in MCF7 breast cancer cells (Fig. 1e). In addition, western blot analysis (Fig. 1f and Supplemental Fig. S7a) confirmed that RBPMS2 and RBM24 protein expression levels were downregulated in hypoxia. On the other hand, FUS protein expression level was upregulated by hypoxia in contrast to mRNA levels that were downregulated. This suggested that although FUS was responsive to hypoxia, its regulation at the mRNA and protein levels differed and more complex mechanisms such as those involving protein translation may be at play. The protein levels of RBM43 protein remained unchanged and therefore the changes in mRNA levels were not consequential within the time frame examined. Although mRNA levels of a fifth splicing regulator MBNL3 also showed decreased expression under hypoxia, the protein expression was not detectable (data not shown) and it may be either degraded or subjected to low translation rates. Since these RNA binding proteins are known to perform prominent functions in the regulation of alternative splicing of pre-mRNAs, it is possible that hypoxic regulation of these splicing factors may account for some of the changes in the alternative splicing landscape of MCF7 cells.

### Acute and chronic phase hypoxia primarily mediates intron retention of alternatively spliced target genes

**Figure 2:**
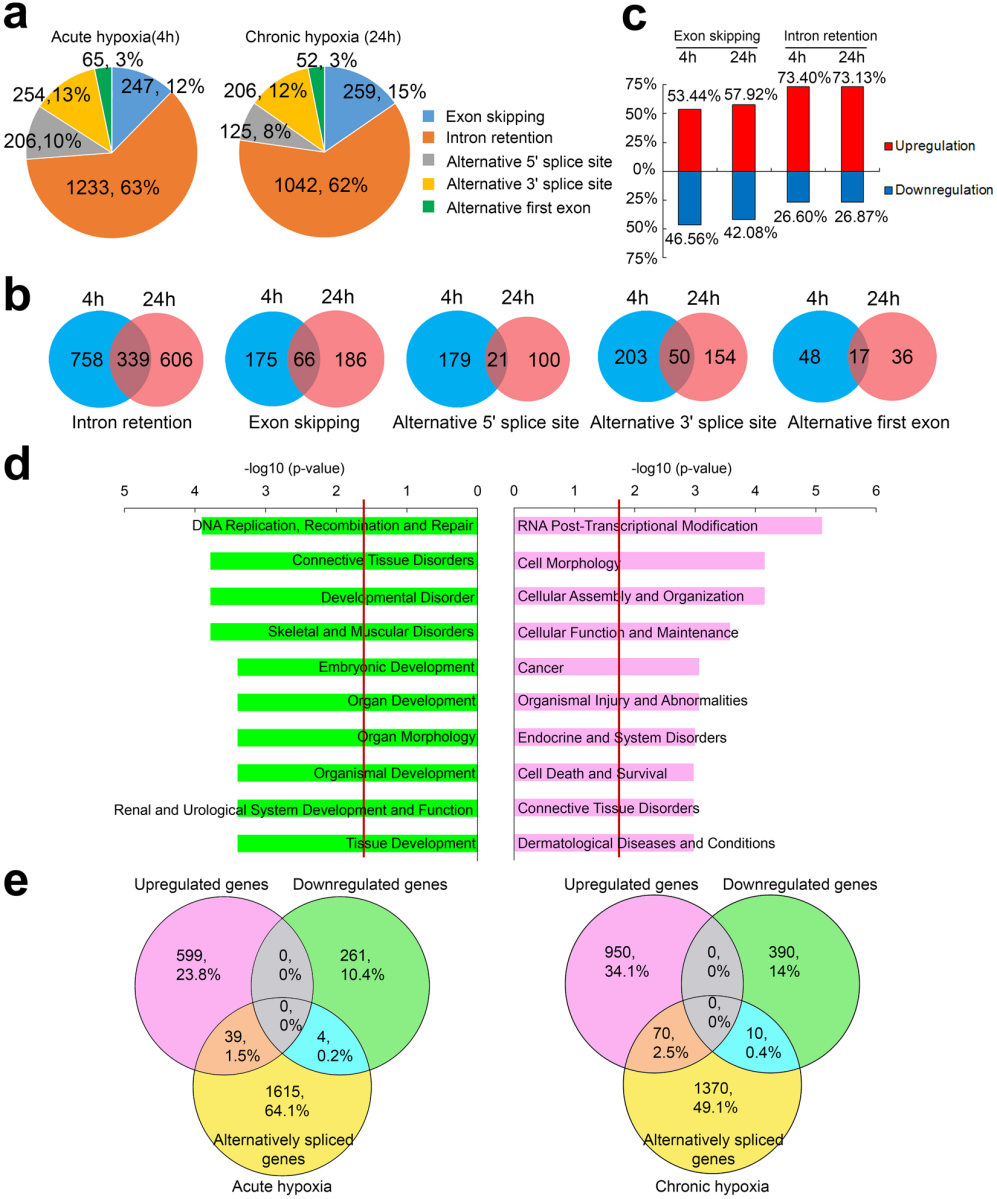
Hypoxia drives alternative splicing in MCF7 cells. (**a**) Pie charts showing the occurrence of 5 classes of alternative splicing events that are induced during acute and chronic hypoxia as identified from n = 1 RNA-Seq experiments. A FDR of < 0.01 and |ΔSI| of ≥ 15% was used as the cutoff. (**b**) Venn diagrams showing the overlap for the 5 types of significantly regulated alternative splicing events during acute and chronic hypoxia. The number of splicing changes and the corresponding percentage out of the total is indicated. (**c**) Bar chart shows the percentage of up- and downregulated exon skipping and intron retention events in acute and chronic hypoxia. **(d)** GO analysis using IPA for significantly down- and upregulated intron retention events (FDR < 0.01 and |ΔSI| ≥ 15%) during chronic hypoxia. Red lines indicate the p-value cutoff of 0.05 for Fisher’s exact test. (**e**) Venn diagrams showing overlaps between genes that are up- or downregulated by at least 1.5-fold and genes undergoing alternative splicing (|ΔSI| ≥ 15%) during acute and chronic hypoxia in MCF7 cells. The number of alternatively spliced genes is less than the number of alternatively spliced events as some genes may be subjected to multiple splicing events.

In addition to the gene expression changes, the RNA-Seq data was used to examine, map and quantitate all splice junctions using the Ensembl database as a reference to determine the extent of alternative splicing in MCF7 cells subjected to normoxia, acute and chronic hypoxia. Changes in the splicing index (ΔSI) were used to measure the difference in the ratio of an alternative spliced mRNA isoform compared to the total isoforms for each gene between cells grown in acute/chronic hypoxic and normoxic conditions. Applying a false discovery rate (FDR) cutoff of 0.01 and |ΔSI| of 15%, we discovered 2005 and 1684 significantly regulated alternative splicing events during acute and chronic hypoxia (Supplementary Table S1). Out of these, 1582 and 1274 splicing events induced during acute and chronic hypoxia were novel and not present in the Ensembl database suggesting that much of the alternative splicing landscape remains unknown and uncharacterized in the breast cancer cells. The alternative splicing events were classified into five types, including exon skipping, intron retention, alternative first exon usage, alternative 5′ splice sites and alternative 3′ splice sites. Intronic retention was the most abundant alternative splicing change (62% in acute and chronic hypoxia), whereas alternative first exon usage was the least common (consistently 3% in acute and chronic hypoxia, Fig. 2a). The ratio of exon skipping, alternative 5′ splice site and alternative 3′ splice site events out of the total number of splicing events was also largely comparable, varying from 8% to 15% in both acute and chronic hypoxia (Fig. 2a) suggesting that the splicing distribution mediated by hypoxia was both reproducible and consistent across different phases. Intriguingly, while the ratios of splicing events were similar, the target genes regulated by acute and chronic hypoxia splicing showed highly disparate overlaps (Fig. 2b). Only approximately 20% (339/1703) of intron retention events were regulated in both acute and chronic hypoxia while the remaining 80% (1364/1703) of genes were regulated uniquely in the acute or chronic phases. Notably, only 7% (21/300) of alternative 5′ splice site events were regulated in both acute and chronic hypoxia while the remaining 93% (279/300) of genes were spliced uniquely in each of these phases. This suggested that the target genes mediated by hypoxia-driven splicing vary in a highly dynamic manner over time across the acute and chronic phases in breast cancer cells. Further analysis of intron retention events which are the most dominant hypoxia-induced splice type, revealed that the upregulation of intron retention comprised 73.40% and 73.13% of total intron retention events at the acute and chronic phases respectively (Fig. 2c). This indicated that hypoxia generally promoted intron retention over intron exclusion in MCF7 cells possibly by the regulation of intron retention factors such as FUS^38^. Similarly, exon skipping dominates over exon inclusion since over half of exon skipping events were upregulated (53.44% in acute hypoxia and 57.92% in chronic hypoxia) compared to exon inclusion (46.56% in acute phase and 42.08% in chronic phase).

**Figure 3:**
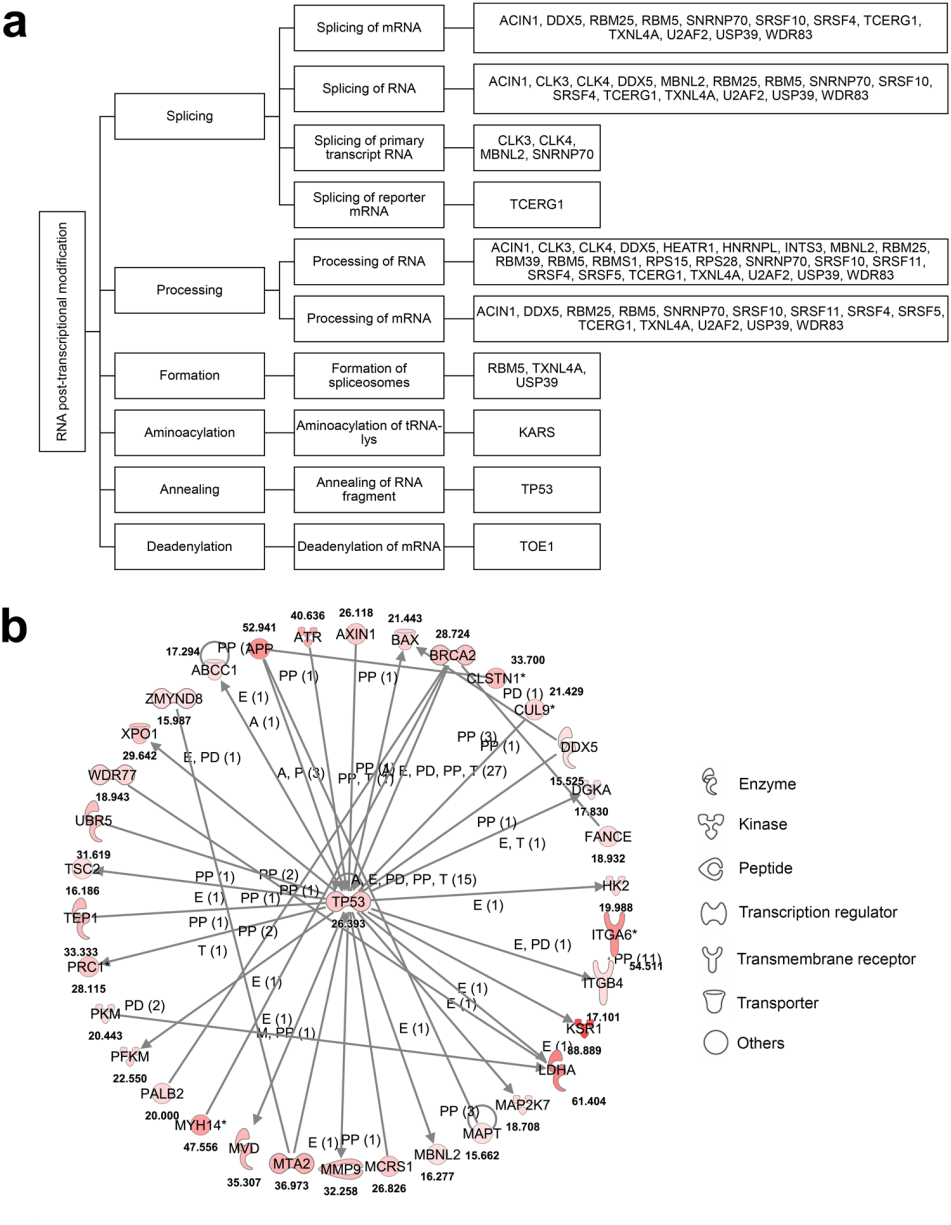
Hypoxia-regulated intron retention targets are involved in RNA post-transcriptional modification and cancer function. (**a**) Tree diagram showing functional distribution of chronic hypoxia-regulated genes with significantly upregulated intron retention events (FDR < 0.01 and |ΔSI| ≥ 15%) that are involved in RNA post-transcriptional modification processes. (**b**) Network of hypoxia-regulated genes with significantly up-regulated intron retention events involved in cancer function. The network was generated using IPA with the criteria that only experimentally observed, direct relationships in human were used. Numbers indicate the ΔSI for each gene. The types of relationships include expression (E), protein-protein interactions (PP), activation (A), transcription (T), protein-DNA interactions (PD) and modifications (M).

To determine whether hypoxia-regulated alternative splicing was enriched in specific cellular functions and disease processes, gene ontology analysis using Ingenuity Pathway Analysis (IPA) was performed on intron retention and exon skipping target genes regulated in acute and chronic hypoxia. We focused in particular, on the intron retention events regulated by chronic hypoxia, since it is the most dominant splice type. The top 10 enriched functions and diseases associated with hypoxia for both up- and downregulated intron retention are shown in the bar charts (Fig. 2d). Genes that undergo downregulated intron retention were involved in DNA replication, recombination and repair, as well as connective tissue, developmental, skeletal and muscular disorders. In contrast, genes involved in RNA post-transcriptional modification, cell morphology, cellular assembly and organization, cellular function and maintenance as well as cancer had upregulated intron retention. Interestingly, some genes subjected to alternative splicing during hypoxia are themselves regulators of the RNA post-transcriptional modification process, including splicing. There was hypoxia-induced intron retention of target genes involved in the processing of RNA, formation of spliceosomes, aminoacylation of tRNA-lys, annealing of RNA fragments and deadenylation of mRNA (Fig. 3a). To determine which of the upregulated intron retention targets were specifically involved in oncogenic processes, we isolated all target genes that had known cancer functions in IPA and constructed a gene network to elucidate their relationship and interactions with each other in the MCF7 breast cancer cells (Fig. 3b). For improved stringency and to generate high confidence results, all connections in the network were limited to experimentally validated findings for direct interactions in human cells and samples. Many important cancer-related genes involved in DNA damage repair (*TP53*, *ATR* and *BRCA2*), apoptosis (*BAX*) and cell metabolism (*PKM, PFKM* and *LDHA*) were subjected to chronic hypoxia-induced intron retention. Interestingly, a number of these cancer-related target genes subjected to hypoxic intron retention were either targets or regulators of TP53. This indicates that much of the hypoxia-induced alternative splicing transcriptome is heavily implicated in the TP53 pathway which is a prominent tumor suppressor pathway in cancer. Therefore hypoxia may be able to mediate oncogenic processes via splicing of TP53-related genes and components.

To further analyze the relationship between hypoxia-regulated gene expression and alternative splicing, we compared genes that are up- and downregulated by at least 1.5-fold and against genes that have a high |ΔSI| splicing index of at least 15% during acute and chronic hypoxia in MCF7 cells. Crucially, we found that during acute hypoxia, only 39/2518 (1.5%) upregulated and 4/2518 (0.2%) downregulated genes also showed significant splicing changes. During chronic hypoxia, this was increased to 70/2790 (2.5%) upregulated and 10/2790 (0.4%) downregulated genes that showed significant splicing changes out of the total set of genes that showed gene expression and/or splicing changes (Fig. 2e). This indicated that hypoxia-regulated mRNA transcription and alternative splicing are primarily independent processes that regulate highly disparate subsets of target genes thus allowing cancer cells to make use of an expanded range of mechanisms for their development and progression.

To validate the accuracy of the RNA-Seq analysis of hypoxia-regulated alternative splicing, a sampling of intron retention, exon skipping and alternative first exon target genes (FDR < 0.01 and |ΔSI| > = 15%) was selected for RT-PCR or qRT-PCR analysis. This selection of candidates included genes showing relatively large splicing alterations, as well as functionally interesting genes with known roles in oncogenicity. MXI1 was examined as a positive control since the alternative first exon usage of MXI1 during hypoxia in MCF7 cells has been previously reported^39^ and this was also confirmed and reproducible in our RNA-Seq analysis (Supplementary Fig. S2b). In total, based on 14 target genes that were validated, we observed a strong concordance (R^2^ = 0.8869) between the quantitative splicing changes measured by RT-PCR or qRT-PCR and RNA-Seq analysis (Supplementary Fig. S2c and d). Therefore, the RNA-Seq analysis was well supported by the independent RT-PCR and qRT-PCR validation for the identity of the target genes that are spliced, as well as the directionality, type and degree of their splicing.

To determine if cellular density has any effects on alternative splicing in the context of hypoxia, we examined the changes in isoform abundance at different plating densities in normoxia and hypoxia (Supplemental Fig. S6a). Isoforms for *LDHA* which were subjected to intron retention, *MARCH7* and *PCBP2* that underwent exon skipping as well as *VGLL4* with alternative first exon usage were quantified by real time qPCR. In all cases examined, cell density did not disrupt relative isoform abundance at comparable densities. Intron retention was upregulated by hypoxia for *LDHA* while the spliced isoform was downregulated. For *MARCH7*, the full length isoform was always more abundant than the **Δ**E9 exon skipping isoform which in turn increases specifically under hypoxia. For *PCBP2*, the exon skipping **Δ**E11 isoform was specifically upregulated during chronic hypoxia while the full length transcript was upregulated by acute hypoxia and then downregulated in the chronic phase. Finally, the *VGLL4-001* isoform was slightly downregulated while *VGLL4-003* was strongly upregulated by both acute and chronic hypoxia. Overall, cell density tends to accentuate the effects of hypoxia-induced changes for all the genes examined either driving up- or downtrends. To determine why this may be the case, we quantified the expression of the canonical hypoxia marker *CA9* as a readout for hypoxic status at different cell densities (Supplemental Fig. S6b). Crucially, cell density had no effect on *CA9* expression in normoxia indicating that the cells were not undergoing hypoxic stress at any point. However, under acute and chronic hypoxia treatments, increasing cell density may lead to more severe hypoxia as indicated by enhanced *CA9* expression. Therefore, while there was sufficient oxygen under normoxia even at high plating densities, under conditions where oxygen is limiting, the increased cell density may result in increased competition for oxygen and more severe hypoxic effects on splicing.

### Hypoxia regulates the intron retention of the LDHA, TNFSF13 and ARHGAP4 metabolic, survival and motility factors

**Figure 4:**
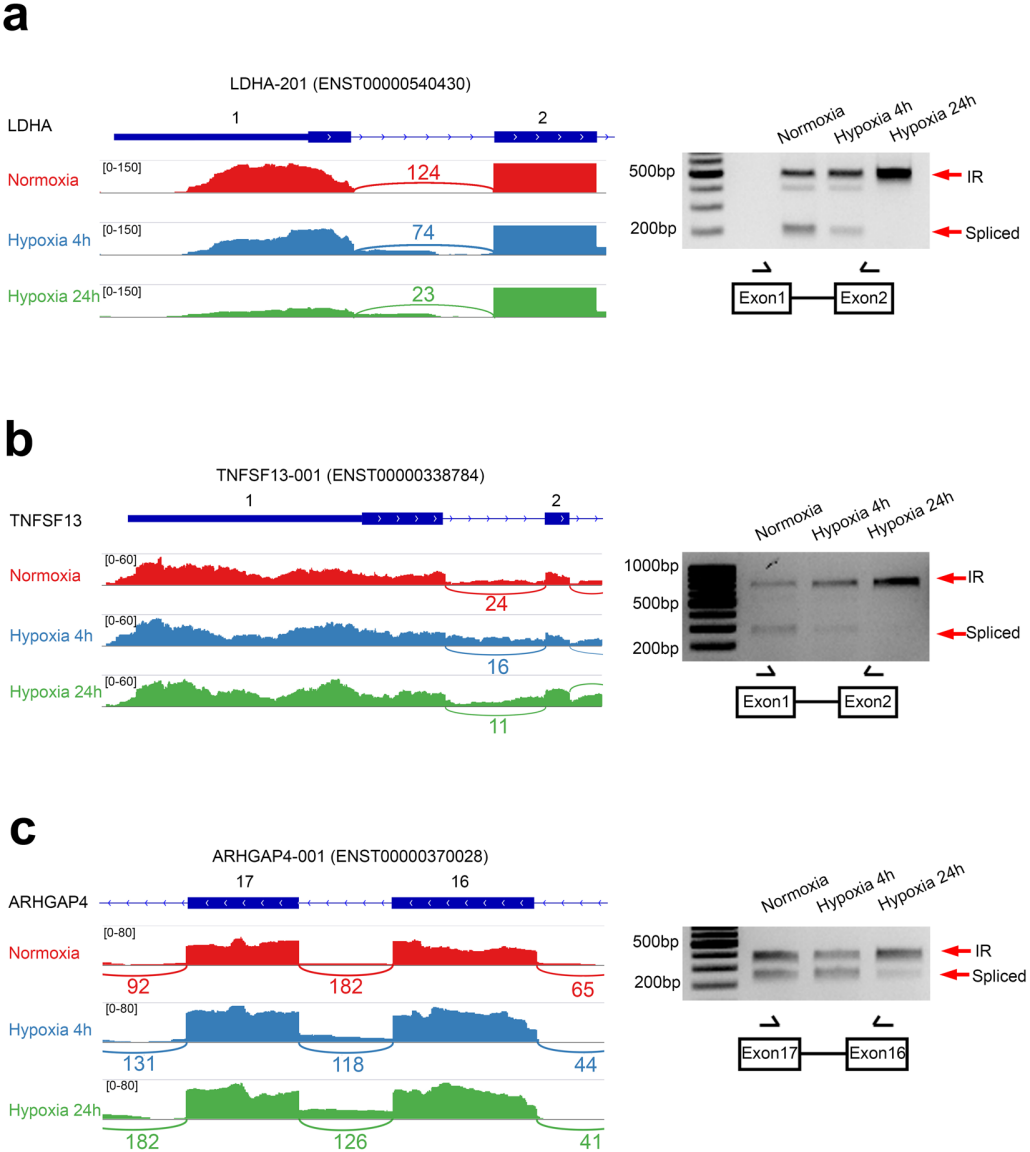
Hypoxia regulates intron retention of *LDHA*, *TNFSF13* and *ARHGAP4* in MCF7 cells. Normalized RNA-Seq reads in normoxia, acute and chronic hypoxia for n = 1 replicate mapped to (**a**) *LDHA*, (**b**) *TNFSF13* and (**c**) *ARHGAP4* were visualized using IGV 2.3 in the left panel. Exon/intron coverage is shown on the same scale for each gene. Numbers indicate exon-exon junction read counts. Representative agarose gels of RT-PCR validation of intron retention events in (**a**) *LDHA*, (**b**) *TNFSF13* and (**c**) *ARHGAP4* are shown on the right. Arrows indicate retained introns (IR, upper bands) or spliced isoforms (Spliced, lower bands). The positions of primers used in the RT-PCR are schematized.

In higher eukaryotes, it has been shown that intron retention is the least common alternative splicing event while exon skipping is more prevalent^40^. Surprisingly, we observed a large number of 1233 and 1042 intron retention events triggered by acute and chronic hypoxia in MCF7 cells (Fig. 2a and Supplementary Table S1). Intron retention can introduce pre-mature stop codons, which lead to NMD or cause truncation of proteins. Therefore, hypoxia-regulated intron retention events may be important for governing protein expression and function. Most of the intron retention events identified in the MCF7 cells are novel and have not been annotated in the Ensembl database (Supplementary Table S1). To confirm the intron retention results arising from the RNA-Seq analysis, we examined 3 target genes *LDHA*, *TNFSF13* and *ARHGAP4* that undergo this splicing type during hypoxia (Fig. 4 and Supplemental Fig. S7b–d). No reverse transcriptase (no RT) PCR controls were also examined to exclude potential genomic DNA contamination and found to be negative (Supplementary Fig. S4a–c).

**Figure 5:**
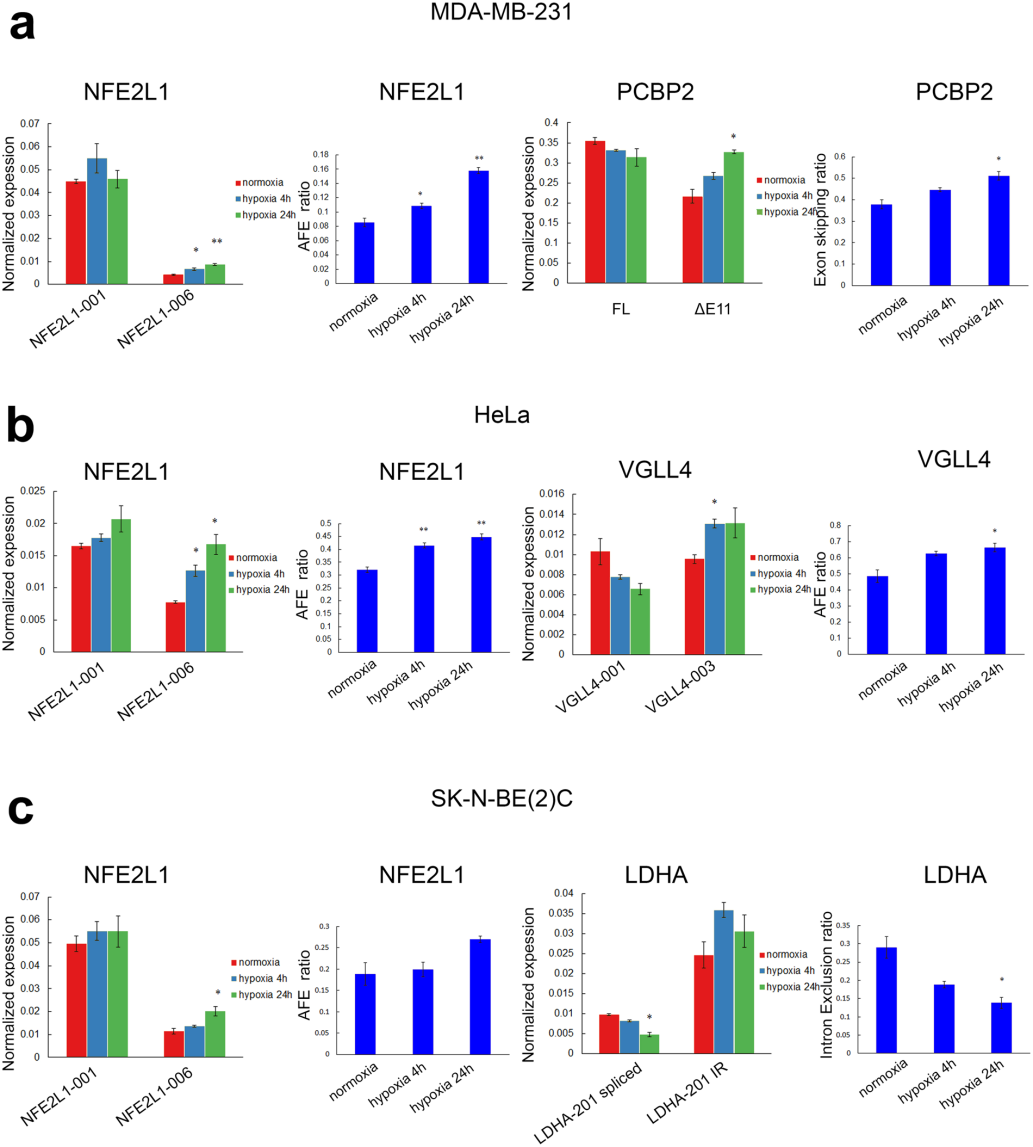
Conservation of hypoxia-induced alternative splicing in MDA-MB-231, HeLa and SK-N-BE(2)C cells. (**a**) Normalized expression of alternative first exon (AFE) usage *NFE2L1* and exon skipping *PCBP2* isoforms in MDA-MB-231 cells was measured by qRT-PCR. (**b**) Normalized expression of AFE *NFE2L1* and *VGLL4* isoforms in HeLa cells was measured by qRT-PCR. (**c**) Normalized expression of AFE NFE2L1 and intron retention LDHA isoforms in SK-N-BE(2)C cells was measured by qRT-PCR. In all 3 cell lines, the relative abundance of alternatively spliced isoforms was calculated as a percentage of the total abundance for all isoforms using the qRT-PCR results (blue bar charts). *PPIA* expression levels were used as the housekeeping reference control. All qRT-PCR analysis was performed with three biological replicates (n = 3). Error bars indicate SEM. Statistical significance was evaluated with Student’s t-test. *indicates p-value < 0.05, **indicates p-value < 0.01.

LDHA (Lactate Dehydrogenase A) is an enzyme that catalyzes the conversion of pyruvate to lactate and produces NAD+, a key metabolic step in anaerobic glycolysis under hypoxia and aerobic glycolysis in cancer cells in what is known as the Warburg effect^41^. *LDHA* is a direct target of HIF1α transcription and its overexpression is correlated with poor prognosis in multiple cancers^42–45^. Multiple *LDHA* isoforms are annotated in Ensembl but they are as yet poorly characterized with unknown functions and modes of regulation. The major isoform expressed in MCF7 cells, *LDHA-001* (ENST00000422447), was significantly induced during acute and chronic hypoxia in MCF7 cells. Interestingly, *LDHA-201* (ENST00000540430) with an alternative first exon compared to *LDHA-001*, was significantly reduced in hypoxia with a corresponding increase in a novel isoform that maintains the retention of intron 1 (Fig. 4a and Supplementary Figs S4a and S7b). Therefore, the hypoxia-induced intronic retention may lead to the loss of the *LDHA-201* isoform. In contrast to *LDHA-001*, *LDHA-201* has an open reading frame starting from the first exon and encodes a larger protein of 361 amino acids and differs from *LDHA-001* in its N-terminus. In the novel intron retention variant of *LDHA-201*, the retained first intron contains multiple stop codons in all three reading frames. These changes in splicing under hypoxia are also largely reflected in SK-N-BE2(C) neuroblastoma cells where the spliced *LDHA-201* isoform is consistently downregulated and the intron retention non-coding *LDHA-201* upregulated under hypoxia compared to the normoxia control (Fig. 5c). The levels of the intron retention isoform increased after cycloheximide (CHX) treatment which inhibits nonsense-mediated decay (NMD, Supplementary Fig. S4d). This indicated that the intron retention isoform was indeed subjected to NMD although this was not sufficient to result in complete loss of the transcript.

TNFSF13 (Tumor Necrosis Factor Superfamily Member 13) encodes a ligand for the TNF receptor family and has known roles in tumor development, adaptive immunity and autoimmune diseases^46, 47^. Hypoxia promoted the retention of the first intron in *TNFSF13* and suppressed the spliced isoform (Fig. 4b and Supplementary Figs S4b and S7c). The intron retention transcripts were not significantly affected by NMD as inhibition of NMD with CHX (Supplementary Fig. S4d) did not increased the isoform levels. Nevertheless, the first intron of *TNFSF13* contains multiple stop codons in all three open reading frames and therefore this transcript may not be translated into protein products. Instead, the expression of the *TNFSF13* transcript isoform that is spliced for intron 1 and encodes the complete protein became suppressed. Since TNFSF13 has an anti-apoptotic role, it is possible that the downregulation of the *TNFSF13* protein-coding transcript by hypoxia-induced intron retention may contribute towards a tumor suppressor effect.

ARHGAP4 (Rho GTPase Activating Protein 4) encodes a protein that catalyzes the hydrolysis of GTP bound to Rho family proteins leading to their inactivation and has been shown to inhibit cell motility^48^. During chronic hypoxia, while the intron 16 retention isoform of *ARHGAP4* was constant compared to normoxia, the spliced intron 16 isoform ARHGAP4-001 (ENST00000370028) that encodes the complete ARHGAP4 protein was significantly suppressed (Fig. 4c and Supplementary Figs S4c and S7d). The CHX treatments did not increase ARHGAP4 intron retention transcript levels (Supplementary Fig. S4d), indicating it was not significantly degraded by NMD. The isoform with intron 16 retention possessed an in-frame pre-mature stop codon that may therefore result in a truncated protein that lacks a complete RhoGAP domain. Consequently, this truncated isoform may not be fully functional compared with the full-length ARHGAP4 protein. Since hypoxia suppressed the expression of the full-length protein coding *ARHGAP4* isoform, it is possible this enhances the motility of MCF7 cells during hypoxia via derepression of Rho family proteins.

### Hypoxia drives exon skipping of MARCH7, PCBP2 and LRCH3

Exon skipping can disrupt the integrity of domain structure, alter domain spacing, introduce pre-mature stop codons and shift the reading frame of the protein. Therefore, regulation of exon skipping in mRNA transcripts plays a critical role in controlling protein expression and determines structure, enzymatic activity, stability as well as interactions with other protein partners. To address if hypoxia is able to mediate exon skipping on the whole transcriptome level, we analyzed the RNA-Seq data on the MCF7 cells with the criteria that the splicing difference |ΔSI| must be ≥15% and FDR <0.01. Subsequently, we identified 247 and 259 exon skipping events that are regulated by acute and chronic hypoxia respectively in MCF7 cells. Comparing the frequency of exon skipping against that of exon inclusion, we found that hypoxia generally promoted exon skipping (132/247 or 53.44% for acute hypoxia and 150/259 or 57.92% for chronic hypoxia). This result is consistent with exon array analysis of Hep3B cells during hypoxia that showed 56% (568/1022) of hypoxia-regulated exon skipping events were favoured^22^. Subsequently, to validate and confirm the exon skipping results in the MCF7 cells, we examined the isoform abundance of *MARCH7*, *PCBP2* and *LRCH3* using RT-PCR or qRT-PCR. The specificity of the exon skipping and exon inclusion primers was confirmed as there were only single PCR products for each primer pair (Supplementary Fig. S5a). Furthermore, the PCR products were cloned and subjected to Sanger sequencing where they were found to contain the correct splice junctions in the correct genes (Supplementary Fig. S5b–d).

**Figure 6:**
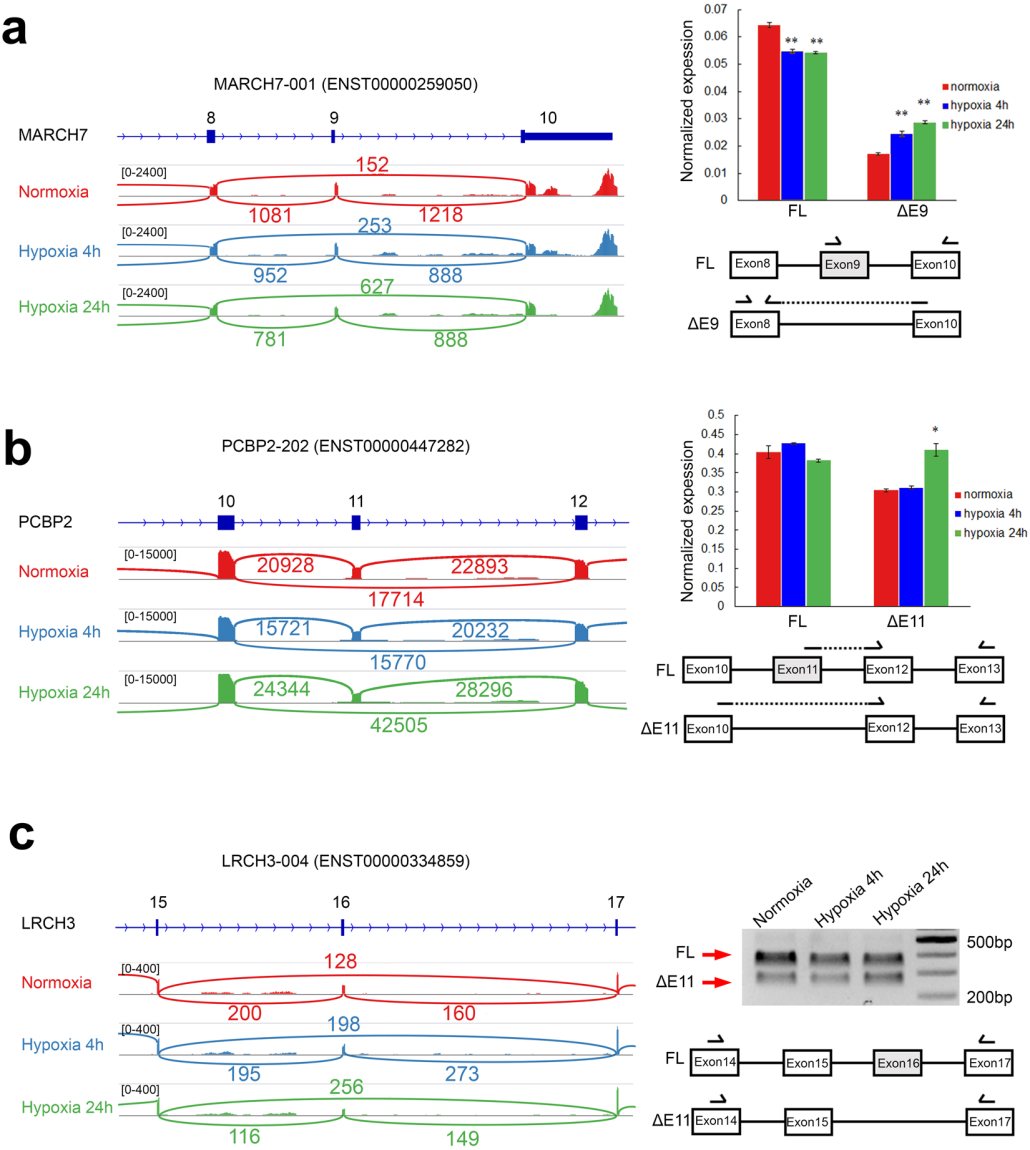
Hypoxia induces exon skipping of *MARCH7*, *PCBP2* and *LRCH3* in MCF7 cells. Normalized RNA-Seq reads in normoxia, acute and chronic hypoxia for n = 1 replicate mapped to (**a**) *MARCH7*, (**b**) *PCBP2* and (**c**) *LRCH3* were displayed using IGV 2.3 in the left panel. Exon and intron coverage is shown on the same scale for each gene. Numbers indicate exon-exon junction read counts. Normalized expression of exon inclusion and exon skipping isoforms for (**a**) *MARCH7* and (**b**) *PCBP2* was measured using qRT-PCR. *PPIA* expression levels were used as the reference housekeeping control. (**c**) Representative agarose gel of RT-PCR validation of *LRCH3* is shown. Arrows indicate the full-length included exon (FL, upper band) and exon skipping (Δ11, lower band) isoforms. The positions of primers used in the RT-PCR and qRT-PCR are schematized. All qRT-PCR and RT-PCR analyses were performed in three biological replicates (n = 3). Error bars indicate SEM. Statistical significance was evaluated with Student’s t-test. *indicates p-value < 0.05, **indicates p-value < 0.01.

MARCH7 (Membrane-Associated Ring Finger (C3HC4) 7) encodes an E3 ubiquitin ligase known to promote ovarian cancer cell proliferation, migration and invasion by regulating NF-κB and Wnt/β-catenin signaling pathways^49^. In MCF7 breast cancer cells, hypoxia slightly downregulated expression of the full-length *MARCH7-001* (ENST00000259050) isoform, but significantly induced the expression of a new isoform where the second last exon 9 has been skipped (Fig. 6a). Skipping of the 49 bp exon 9 causes a shift in the open reading frame of the last exon. As a result, the protein encoded by the skipped exon 9 isoform contains the same RING-CH domain, but has a different C-terminus. It remains unclear what is the function of this new exon 9 skipping isoform. However, it is possible that this isoform may have different activities or interactions from full-length MARCH7 in the context of canonical proliferative and migration functions.

PCBP2 (Poly(RC) Binding Protein 2) encodes for a protein that contains three K homology (KH) domains involved in RNA binding^50, 51^. PCBP2 also acts as an iron chaperone for PHD proteins, hence depletion of PCBP2 could suppress PHD activity and rescue prolyl hydroxylation and proteasome degradation of HIF-1α resulting in its accumulation^52^. We found that hypoxia induced exon 11 skipping of *PCBP2-202* (ENST00000447282) in MCF7 cells (Fig. 6b), and this was also reproducible in the highly metastatic triple negative MDA-MB-231 breast cancer cells (Fig. 5a). The skipping of exon 11 leads to a 13 amino acid shortening of the linker region between the second and third KH domains. Therefore, it is possible that this could affect PCBP2 function in the stabilization of its RNA targets.

LRCH3 (Leucine-Rich Repeats and Calponin Homology (CH) Domain Containing 3) encodes a protein with multiple leucine-rich repeats (LRR) in the N-terminus and a calponin homology (CH) domain in the C-terminus. It has been shown that LRCH proteins are cytoskeletal organizers during the division of Drosophila cells^53^. Hypoxia significantly increased the skipping of exon 15 of *LRCH3-004* (ENST00000334859) in MCF7 cells (Fig. 6c and Supplemental Fig. S7e). Exon 15 of *LRCH3-004* is 72 bp long and its skipping causes a deletion of 24 amino acids between the leucine-rich repeats and calponin homology domain of the LRCH3 protein. This change in domain spacing may therefore affect the ability of the LRR and CH domains to interact with other proteins with possible consequences on MCF7 cytoskeletal organization.

### Hypoxia promotes alternative first exon usage of VGLL4, AHNAK and NFE2L1

Although alternative first exon usage was the least frequent amongst the five types of splicing induced by hypoxia, it may be directed by HIF activation itself under hypoxia. Previous studies have shown that HIFs can indeed regulate the expression of their target genes through alternative promoter usage. MXI1 is a component of the oncogenic MYC pathway where it acts as a negative regulator of MYC transcription via competition for the MAX copartner^54^. Acute hypoxia rapidly induced the expression of the *MXI1-004* isoform, while the *MXI1-006* isoform which differs from *MXI1-004* in the first exon only increases in chronic hypoxia^39^. Therefore, it is also possible that some target genes are similarly regulated by alternative first exon usage in MCF7 breast cancer cells under hypoxia. To validate the alternative first exon events in the MCF7 cells identified from the RNA-Seq analysis, we examined the isoforms induced during hypoxia by qRT-PCR for the target genes *VGLL4*, *AHNAK* and *NFE2L1*.

**Figure 7:**
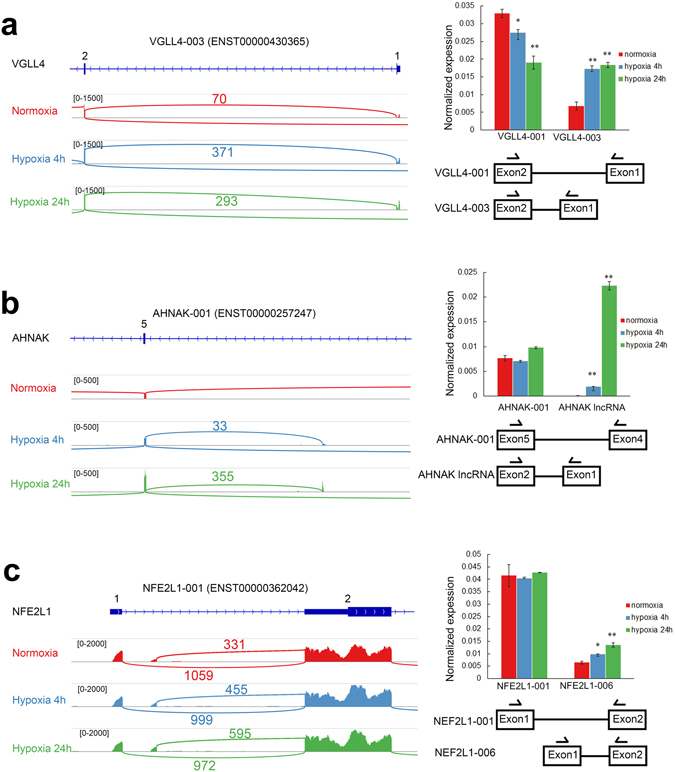
Hypoxia drives alternative first exon usage of *VGLL4*, *AHNAK* and *NFE2L1* in MCF7 cells. Normalized RNA-Seq reads in normoxia, acute and chronic hypoxia for n = 1 replicate mapped to (**a**) *VGLL4*, (**b**) *AHNAK* and (**c**) *NFE2L1* were displayed using IGV 2.3 in the sashimi plots (left panel). Exon coverage is shown on the same scale for each gene. Numbers indicate exon-exon junction counts. The normalized expression of the alternative first exon isoforms for (**a**) *VGLL4*, (**b**) *AHNAK* and **(c)** *NFE2L1* was measured using qRT-PCR. *PPIA* expression was used as the housekeeping reference control. The positions of primers used in the qRT-PCR are schematized. All qRT-PCR analysis was performed in three biological (n = 3). Error bars indicate SEM. Statistical significance was evaluated with Student’s t-test. *indicates p-value < 0.05, **indicates p-value < 0.01

VGLL4 (Vestigial-Like Family Member 4) is a transcriptional cofactor which functions as a tumor suppressor in multiple cancers^55–57^. Here, we detected two isoforms of *VGLL4*, *VGLL4-001* (ENST00000273038) and *VGLL4-003* (ENST00000430365) in MCF7 breast cancer cells. *VGLL4-001* consists of 6 exons and is translated from the second exon, while *VGLL4-003* consists of 5 exons and is translated from the first exon. Apart from the unique first exon, *VGLL4-003* shares the same last 4 exons with *VGLL4-001* and therefore, the proteins encoded by the two *VGLL4* variants differ in their N-termini. Hypoxia suppressed the expression of *VGLL4-001* by up to 1.5-fold and induced *VGLL4-003* significantly by up to 3-fold in MCF7 cells (Fig. 7a). Similar results were also obtained in HeLa cervical cancer cells although to a lesser degree where *VGLL4-003* was upregulated by 1.5-fold and *VGLL4-001* was downregulated by 1.5-fold (Fig. 5b). This difference in the N-termini of the two VGLL4 isoforms may lead to changes in protein stability, localization and interaction with other cofactors with possible consequences on VGLL4 tumor suppressor function.

AHNAK (AHNAK Nucleoprotein or Desmoyokin) encodes a large 700 kDa protein involved in calcium channel function, actin cytoskeleton organization and tumor metastasis^58–61^. From the RNA-Seq analysis of the MCF7 cells, we found that 3 isoforms of AHNAK are expressed. Besides the large *AHNAK-005* (ENST00000378024) isoform that corresponds to the 700 kDa protein, a shorter isoform *AHNAK-001* (ENST00000257247) was also identified that shares the first 4 exons with *AHNAK-005*. Furthermore, the *AHNAK-001* isoform contains two additional exons in place of the large exon 5 (18,170 bp) found in the large isoform. *AHNAK-001* remains in-frame and encodes a small protein of 17 kDa where it has been shown to be able to regulate the splicing of its own locus^62^. Interestingly, we identified a novel third isoform of AHNAK, which was highly induced by 240-fold during hypoxia as determined by qPCR validation (Fig. 7b). To confirm that alternative first exon usage of AHNAK has indeed taken place, we performed 5′ RACE followed by Sanger sequencing of the RACE product. Structurally, we found that the novel isoform had a unique first exon, while sharing the same last two exons as the short *AHNAK-001* isoform as confirmed by 3′ RACE (Supplementary Fig. S3). This isoform is 700 bp long with no open reading frames making it a hypoxia-induced lncRNA that may have additional effects beyond what is known of canonical AHNAK function.

NFE2L1 (Nuclear Factor, Erythroid 2 Like 1) encodes a transcription factor involved in genetic instability and hepatic neoplasia^63–65^. Here, we show that the promoter usage of different *NFE2L1* first exons that correspond to the 5′ UTR is significantly altered in MCF7 cells in hypoxia. Hypoxia specifically induced the expression of the first exon corresponding to the *NFE2L1-006* (ENST00000361665) isoform by 2-fold but has no significant effects on the expression of *NFE2L1-001* (ENST00000362042) in MCF7 cells (Fig. 7c). The specific induction of the *NFE2L1-006* isoform is highly conserved, as similar inductions were obtained in HeLa (upregulated by up to 2-fold), triple negative breast cancer MDA-MB-231 (2-fold) and SK-N-BE(2)C neuroblastoma cells (1.7-fold, Fig. 5a–c). Although the alternative first exons correspond to the non-coding 5′UTR region, differences in 5′ UTR sequences are known to affect translation efficiency^66, 67^ and therefore may be important for the regulation of NFE2L1 protein abundance.

## Discussion

Dysregulation of alternative splicing is one of the key features of cancer^68^. Aberrant splicing of many genes is known to promote cancer cell proliferation, survival, migration, invasion and metastasis^68, 69^. Hypoxia, as a common microenvironmental parameter in most solid tumors, also governs alternative splicing extensively in breast cancer cells as shown in this study. As a result, the alternatively spliced transcriptome may allow further avenues for cancer cells to overcome the initial checkpoints imposed by low oxygen availability subsequently leading to disease progression and the development of a more aggressive phenotype. Previous studies on hypoxia-regulated alternative splicing in breast cancer cells have been limited to single gene examples such as for *CYR61* and *CD44* ^19, 20^ while the only cancer-specific genome-wide study has been an exon array analysis in hepatoma Hep3B cells^22^. Exon arrays rely on annotated gene databases for probe design and may be unable to detect novel splicing events, such as novel skipped exons and alternative first exons^70^. Here we used deep RNA sequencing technology that does not rely on prior knowledge or information to analyze the global effects of hypoxia on alternative splicing in breast cancer cells providing a comprehensive and detailed picture of hypoxic regulation of alternative splicing. We demonstrated that acute and chronic hypoxia induces 2005 and 1684 alternative splicing events even at a high stringency FDR cutoff of 0.01, of which 1582 and 1274 are novel splicing events that are not present in the Ensembl database. This indicates that the splicing transcriptome remains largely unknown and demonstrates the utility of RNA-Seq in the unbiased detection of novel splicing variants.

Furthermore, in contrast to the Hep3B exon array studies in which intron retention constitutes only 11% of total hypoxia-induced splicing events while exon skipping was the most common at 51%^22^, our study showed that intron retention was the most prominent hypoxia-induced splicing event in breast cancer cells (62% in both acute and chronic hypoxia) while exon skipping was in the minority (12% and 15% in acute and chronic hypoxia). This may be due to the intrinsic design limitation of exon arrays to probes that map predominantly to exons and lack information on introns therefore accounting for a preponderance of exonic splicing events and lower detection of intronic events. Intron retention is the most abundant type of alternative splicing in lower metazoans and the rarest type in vertebrates and invertebrates^40, 71, 72^. Therefore, intron retention has been relegated as a form of mis-splicing^40^. However, the regulatory importance of intronic retention can be seen in how it affects the expression of canonical transcripts through NMD via the introduction of premature stop codons. In addition, intron retention can also produce truncated protein isoforms that may differ from full-length isoforms in terms of function, cellular localization, enzymatic activity, stability and binding with protein partners^73–75^. It is therefore possible that the hypoxic mediation of intronic retention in breast cancer cells can also have important consequences on these processes.

Our RNA-Seq results indicate that genes such as *VGLL4*, *AHNAK* and *NFE2L1* are subjected to alternative first exon usage under hypoxia. When examined in other datasets, these genes were also found to contain HIF1A or HIF2 binding sites. In ChIP-Seq experiments, *VGLL4* was shown to contain a HIF1A binding site at chr3:11658884–11659327 although no HRE was found within this region^76^. In the same study, *AHNAK* was identified to contain a HIF1A binding site at chr11:62024325–62024606 (HRE chr11:62024566) as well as a HIF2A binding site at chr11:62024508–62024696 (HRE chr11:62024566 and chr11:62024605)^76^. In a separate ChIP-chip study, *NFE2L1* was shown to contain a HIF1A and HIF2A binding site at chr17:43481265–43481686 with two HREs at chr17:43481343 and chr17:43481611^77^. Therefore it is possible that preferential selection and differential regulation of these HREs, HIF binding sites or other response elements under hypoxia may account for the alternative first exon usage.

From comparisons of the alternative splicing of MCF7 cells with other cancer cell lines, we found that this is highly cell-type specific upon hypoxic induction. Many of the hypoxia-induced splicing we found in MCF7 cells did not occur in the other cancer cell lines. The novel lncRNA isoform of *AHNAK* could not be detected in HeLa, MDA-MB-231 or SK-N-BE(2)C cells (data not shown) and is therefore specific to MCF7 where it was strongly upregulated by 240-fold. Hypoxia-induced exon skipping of *PCBP2* was also found in MDA-MB-231 cells but not in HeLa and SK-N-BE(2)C cells and therefore could be more prevalent in breast cancer cells. Loss and reduction of the intron 1 spliced isoform *LDHA-201* was observed only in MCF7 and SK-N-BE(2)C but not in HeLa and MDA-MB-231 cells. In contrast, we determined that induction of the specific *NFE2L1-006* isoform was well conserved in MCF7, HeLa, MDA-MB-231 and SK-N-BE(2)C cells. Hence, alternative splicing mediated by hypoxia has both cell-type specific as well as highly conserved universal target genes. When comparing our study to previous studies on endothelial and Hep3B liver cancer cells^21, 22^, none of the genes validated from their datasets showed significant splicing changes in our study. This indicates that hypoxia-induced alternative splicing has predominantly different targets in different cell types with possibly only a minority of highly conserved universal splicing targets. The conserved hypoxia-induced alternative splicing events identified in our study, such as for *NFE2L1* in MCF7, HeLa, MDA-MB-231 and SK-N-BE(2)C cells may indicate that they are critical for hypoxia function and response.

Previous studies on the impact of hypoxia on the transcriptome were focused on changes in gene expression patterns and pathways regulated by canonical hypoxia-induced genes. Here we showed that hypoxia-regulated alternative splicing presents an additional layer of control in the transcriptome. It is known that hypoxia impairs double strand breaks and mismatch repair pathways^78^. In our study, we found that genes with downregulated intron retention were also significantly enriched in DNA damage and repair pathways. This indicates that hypoxia may modulate DNA damage and repair processes not only through transcriptional regulation, but also through post-transcriptional alternative splicing. Similarly, we found many cellular processes were regulated both transcriptionally and post-transcriptionally under hypoxia, including carbohydrate metabolism, cellular movement, growth and proliferative, cell cycle, cell death and survival. These cellular processes are critical for cancer cells to survive and proliferate in the hypoxic microenvironment. The concerted regulation in transcription and alternative splicing may work synergistically on multiple cellular processes facilitating the adaptation of cancer cells to hypoxia. However, we also found that some cellular processes were subjected exclusively to regulation by alternative splicing but not by changes in gene expression. Genes with upregulation of intron retention in chronic hypoxia were enriched in RNA post-transcriptional modification processes, but the same analysis of hypoxia-regulated gene expression did not show that RNA post-transcriptional modification process was significantly affected by hypoxia. Therefore, there are cellular processes occurring in the cancer cells examined that are regulated only by hypoxia-induced alternative splicing with as yet uncharacterized roles in oncogenesis.

In conclusion, our RNA-Seq analysis revealed the global gene expression and alternative splicing changes regulated by hypoxia in breast cancer cells. Based on the RNA-Seq analysis we confirmed the splicing of genes that have known roles in cancer function. By studying the hypoxia-induced alternative splicing landscape, this offers novel insights into further mechanisms by which hypoxia is able to induce the adaptation of cancer cells under conditions of low oxygen availability commonly occurring in tumors.

## Methods

### Cell culture

Human breast cancer MCF7 (ER+, PR+, HER2-, HTB-22) and MDA-MB-231 (triple negative, HTB-26), cervical cancer HeLa and neuroblastoma SKN-BE-2(C) (CRL-2268) cell lines were obtained from ATCC and maintained in DMEM (Nacalai Tesque), supplemented with 10% fetal bovine serum (Sigma-Aldrich), 100 mM non-essential amino acids, 100 U/ml penicillin, 100 mg/ml streptomycin, 2 mM GlutaMAX-I (Invitrogen) and 55 mM β-mercaptoethanol (Sigma) in a humidified incubator at 37 °C with 5% CO_2_. MCF7, MDA-MB-231 and HeLa cells were authenticated in 2014 and SK-N-BE(2)C in 2016 using the GenePrint 10 System (Promega). Mycoplasma testing was conducted routinely for each qRT-PCR run using the forward GGGAGCAAACAGGATTAGATACCCT and reverse TGCACCATGTGTCACTCTGTTAACCTC primers that detect mycoplasma 16 S rRNA. For hypoxia treatments, these were carried out in 1% O_2_ and 5% CO_2_ at 37 °C in an Invivo2 400 hypoxia workstation (Ruskinn Technology). For all cell lines used, 0.6 × 10^6^ cells were seeded simultaneously into 6 cm tissue culture dishes and all culturing conditions were carried out in parallel in the same 24 h window. For the acute phase, cells were cultured for 20 h followed by 4 h of hypoxia while chronic treatments consist of continuous culture for 24 h in hypoxia. Normoxia controls were carried out simultaneously for 24 h in 21% O_2_ and 5% CO_2_ at 37 °C in a Forma Steri-Cycle CO_2_ incubator (Thermo Fisher Scientific). For the cell density experiments, MCF7 cells were seeded at a density of 0.15, 0.3, 0.6 and 1.2 million cells in 6 cm tissue culture dishes and treated with normoxia and hypoxia as described above.

### Western blot

Cells were washed with PBS and M-PER protein lysis buffer with cOmplete™ Protease Inhibitor (Roche) was used to harvest the protein. Cell lysates were collected in 1.5 ml Eppendorf tubes and centrifuged at 12,000 g for 10 minutes at 4 °C to remove insoluble debris. Bradford assay (Bio-Rad) was performed to measure the protein concentration. After dilution in 4X loading dye and heating at 95 °C for 10 min, protein samples of equal amount were subjected to SDS-PAGE and transferred to PVDF membranes. Membranes were incubated with anti-RBM43 (ab181026, Abcam), Anti-FUS (ab23439, Abcam), Anti-RBPMS2 (EPR13121-79, Abcam), Anti-RBM24 (ab94567, Abcam) and anti-actin (I-19, Santa Cruz) antibodies at 4 °C overnight. Primary antibodies were detected with anti-mouse horseradish peroxidase-coupled secondary antibodies.

### Apoptosis assay

MCF-7 cells were seeded in 6 well plates at 0.3 × 10^6^ cells per well and allowed to grow overnight. 24 h normoxia, 20 h normoxia followed by 4 h hypoxia and 24 h hypoxia treatments were administered. Cells were dissociated from culture plates using 1 ml TrypLE (Thermo Scientific). Cells were washed with Annexin V binding buffer (BD Pharmingen) and stained with Annexin V Alexa Fluor 647 conjugate and SYTOX Blue at 1:1000 each (Thermo Scientific) prior to analysis using the LSR II Flow Cytometry System (BD Pharmingen).

### RT-PCR and qRT-PCR validation

Total RNA was extracted from cells using the RNeasy Mini kit (Qiagen). Subsequently, cDNA was synthesized from 1 μg of total RNA using the iScript cDNA Synthesis Kit (Bio-Rad). RT-PCR was performed using DreamTaq DNA Polymerase (Life Technologies). PCR cycling conditions include a 5 min initial denaturation at 95 °C, followed by 30–35 cycles of 95 °C denaturation for 30 sec, 58 °C annealing step for 30 sec and 72 °C extension for 30 sec. PCR products were subjected to agarose gel electrophoresis in TAE buffer (40 mM Tris-Acetate, 1 mM EDTA, pH 8.0), stained with SYBR Green I Nucleic Acid Gel Stain (Invitrogen), visualized and quantified using ImageJ^79^. Real-time qPCR was performed using the KAPA SYBR FAST qPCR kit (Kapa Biosystems) on an ABI 7900HT Real-Time PCR System (Applied Biosystems) according to the manufacturer’s instructions. All experiments were performed using 3 biological replicates. Sequences of primers used for gene expression are shown in Supplementary Table S3. For specificity of the exon-exon junction primers, RT-PCR products were gel extracted and TA cloned into vectors before being subjected to Sanger sequencing. For 5′ and 3′ RACE-PCR, this was carried out using the SMARTer® RACE 5′/3′ Kit (Clontech) according to the manufacturer’s protocol. The AHNAK specific 5′ RACE reverse primer is CCAGTGCTGATGGCTGTGGTGTGT and the 3′ RACE forward primer is AAGCGGCCAGGAAGAAAACCACCCC. The PCR product was cloned into pJET vectors and sequenced to obtain the full length AHNAK transcript sequence.

### RNA-Sequencing analysis

MCF7 cells were subjected to 4 h and 24 h hypoxia treatment with a 24 h normoxia control. RNA extraction was carried out using the RNeasy Mini kit and subjected to 90 bp paired-end sequencing (BGI). Read mapping was conducted using STAR^80^ with the human genome issue 19 (hg19) as the annotation library. Read statistics are summarized in Supplementary Table S2. The RNA-Seq data was deposited in NCBI GEO with the accession number GSE97317.

For the analysis of MCF7 splicing, this was carried out based on our previous method^81^ with the following modifications. Mapped reads were further filtered and classified into the following three groups based on their distance and location around annotated splice sites: 1) reads that mapped to exon-exon junctions, 2) reads bridging exon-intron junctions, and 3) reads that mapped completely to introns. The reads in Group 3 were not used for the identification of differential splicing candidates, but for intron retention coverage and depth calculations. The reads in Groups 1 and 2 were used for the calculation of the splicing index (SI) for each splice site for the selection of potential differential splicing candidates. To identify the various types of splicing events, the SI calculation was adjusted from type to type (Supplementary Fig. S1). Next, all potential splicing candidates were classified into either known/annotated alternative splicing or novel splicing events that are not represented in the annotation library, and were evaluated with p values and differences in the SI. For this study, only candidates with |ΔSI| ≥ 15% and FDR < 0.01 were defined as significant differentially spliced candidates.

Heatmaps were generated using Cluster and TreeView^82^ while Venn diagrams were produced using Venny (*Oliveros, J.C. (2007*–*2015) Venny. An interactive tool for comparing lists with Venn diagrams*. http://bioinfogp.cnb.csic.es/tools/venny/index.html). For analysis of genes, their functional relationships and network generation, the Ingenuity Pathway Analysis platform (IPA, Qiagen) was used. Significant associations with functional categories were identified using Fisher’s exact test at a p-value threshold of <0.05.

## Electronic supplementary material

Supplementary Information

## References

[1] Höckel M, Schlenger K, Höckel S, Vaupel P (1999). Hypoxic cervical cancers with low apoptotic index are highly aggressive. Cancer research.

[2] Höckel M, Vaupel P (2001). Tumor hypoxia: definitions and current clinical, biologic, and molecular aspects. Journal of the National Cancer Institute.

[3] Chan DA, Krieg AJ, Turcotte S, Giaccia AJ (2007). HIF gene expression in cancer therapy. Methods in enzymology.

[4] Denko NC (2008). Hypoxia, HIF1 and glucose metabolism in the solid tumour. Nature Reviews Cancer.

[5] Harris AL (2002). Hypoxia—a key regulatory factor in tumour growth. Nature Reviews Cancer.

[6] Brahimi-Horn MC, Chiche J, Pouysségur J (2007). Hypoxia and cancer. Journal of molecular medicine.

[7] Tanimoto K, Makino Y, Pereira T, Poellinger L (2000). Mechanism of regulation of the hypoxia‐inducible factor‐1α by the von Hippel‐Lindau tumor suppressor protein. The EMBO journal.

[8] Cockman ME (2000). Hypoxia inducible factor-α binding and ubiquitylation by the von Hippel-Lindau tumor suppressor protein. Journal of Biological Chemistry.

[9] Jaakkola P (2001). Targeting of HIF-alpha to the von Hippel-Lindau ubiquitylation complex by O2-regulated prolyl hydroxylation. Science (New York, N.Y.).

[10] Ivan M (2001). HIFα targeted for VHL-mediated destruction by proline hydroxylation: implications for O2 sensing. Science (New York, N.Y.).

[11] Kallio PJ, Pongratz I, Gradin K, McGuire J, Poellinger L (1997). Activation of hypoxia-inducible factor 1α: posttranscriptional regulation and conformational change by recruitment of the Arnt transcription factor. Proceedings of the National Academy of Sciences.

[12] Ruas JL, Poellinger L, Pereira T (2005). Role of CBP in regulating HIF-1-mediated activation of transcription. Journal of cell science.

[13] Lendahl U, Lee KL, Yang H, Poellinger L (2009). Generating specificity and diversity in the transcriptional response to hypoxia. Nature Reviews Genetics.

[14] Chi J-T (2006). Gene expression programs in response to hypoxia: cell type specificity and prognostic significance in human cancers. PLoS Med.

[15] Benita Y (2009). An integrative genomics approach identifies Hypoxia Inducible Factor-1 (HIF-1)-target genes that form the core response to hypoxia. Nucleic acids research.

[16] Pan Q, Shai O, Lee LJ, Frey BJ, Blencowe BJ (2008). Deep surveying of alternative splicing complexity in the human transcriptome by high-throughput sequencing. Nature genetics.

[17] Lewis BP, Green RE, Brenner SE (2003). Evidence for the widespread coupling of alternative splicing and nonsense-mediated mRNA decay in humans. Proceedings of the National Academy of Sciences.

[18] Xie D (2004). Levels of expression of CYR61 and CTGF are prognostic for tumor progression and survival of individuals with gliomas. Clinical Cancer Research.

[19] Hirschfeld M, zur Hausen A, Bettendorf H, Jäger M, Stickeler E (2009). Alternative splicing of Cyr61 is regulated by hypoxia and significantly changed in breast cancer. Cancer research.

[20] Krishnamachary B (2012). Hypoxia regulates CD44 and its variant isoforms through HIF-1α in triple negative breast cancer. PLoS One.

[21] Weigand JE, Boeckel J-N, Gellert P, Dimmeler S (2012). Hypoxia-induced alternative splicing in endothelial cells. PloS one.

[22] Sena JA, Wang L, Heasley LE, Hu C-J (2014). Hypoxia Regulates Alternative Splicing of HIF and non-HIF Target Genes. Molecular Cancer Research.

[23] Yao Y (2016). Global profiling of the gene expression and alternative splicing events during hypoxia-regulated chondrogenic differentiation in human cartilage endplate-derived stem cells. Genomics.

[24] Knowles HJ, Harris AL (2001). Hypoxia and oxidative stress in breast cancer. Hypoxia and tumourigenesis. Breast Cancer Res.

[25] Brown NS, Bicknell R (2001). Hypoxia and oxidative stress in breast cancer. Oxidative stress: its effects on the growth, metastatic potential and response to therapy of breast cancer. Breast Cancer Res.

[26] Vleugel M (2005). Differential prognostic impact of hypoxia induced and diffuse HIF-1α expression in invasive breast cancer. Journal of clinical pathology.

[27] Lundgren K, Holm C, Landberg G (2007). Hypoxia and breast cancer: prognostic and therapeutic implications. Cell Mol Life Sci.

[28] Chia SK (2001). Prognostic significance of a novel hypoxia-regulated marker, carbonic anhydrase IX, in invasive breast carcinoma. Journal of clinical oncology: official journal of the American Society of Clinical Oncology.

[29] Dales JP (2005). Overexpression of hypoxia‐inducible factor HIF‐1α predicts early relapse in breast cancer: Retrospective study in a series of 745 patients. International Journal of Cancer.

[30] Chen J, Imanaka N, Griffin J (2010). Hypoxia potentiates Notch signaling in breast cancer leading to decreased E-cadherin expression and increased cell migration and invasion. British journal of cancer.

[31] Munoz-Najar U, Neurath K, Vumbaca F, Claffey K (2006). Hypoxia stimulates breast carcinoma cell invasion through MT1-MMP and MMP-2 activation. Oncogene.

[32] Yoon S-O, Shin S, Mercurio AM (2005). Hypoxia stimulates carcinoma invasion by stabilizing microtubules and promoting the Rab11 trafficking of the α6β4 integrin. Cancer research.

[33] Schoppmann SF (2006). Hypoxia inducible factor-1α correlates with VEGF-C expression and lymphangiogenesis in breast cancer. Breast cancer research and treatment.

[34] Holmquist-Mengelbier L (2006). Recruitment of HIF-1alpha and HIF-2alpha to common target genes is differentially regulated in neuroblastoma: HIF-2alpha promotes an aggressive phenotype. Cancer cell.

[35] Ishigaki, S. *et al*. Position-dependent FUS-RNA interactions regulate alternative splicing events and transcriptions. *Scientific reports* **2** (2012). 10.1038/srep00529 PMC3402842 22829983

[36] Yang J (2014). RBM24 is a major regulator of muscle-specific alternative splicing. Developmental cell.

[37] Zhang, T. *et al*. Rbm24 Regulates Alternative Splicing Switch in Embryonic Stem Cell Cardiac Lineage Differentiation. *STEM CELLS* (2016). 10.1002/stem.2366 26990106

[38] Reber S (2016). Minor intron splicing is regulated by FUS and affected by ALS-associated FUS mutants. EMBO J.

[39] Löfstedt T (2009). HIF-1α induces MXI1 by alternate promoter usage in human neuroblastoma cells. Experimental cell research.

[40] Kim E, Magen A, Ast G (2007). Different levels of alternative splicing among eukaryotes. Nucleic acids research.

[41] Vander Heiden MG, Cantley LC, Thompson CB (2009). Understanding the Warburg effect: the metabolic requirements of cell proliferation. Science (New York, N.Y.).

[42] Goldman RD, Kaplan NO, Hall TC (1964). Lactic dehydrogenase in human neoplastic tissues. Cancer research.

[43] Semenza GL (1996). Hypoxia response elements in the aldolase A, enolase 1, and lactate dehydrogenase A gene promoters contain essential binding sites for hypoxia-inducible factor 1. Journal of Biological Chemistry.

[44] Koukourakis MI, Giatromanolaki A, Simopoulos C, Polychronidis A, Sivridis E (2005). Lactate dehydrogenase 5 (LDH5) relates to up-regulated hypoxia inducible factor pathway and metastasis in colorectal cancer. Clinical & experimental metastasis.

[45] Koukourakis M (2003). Lactate dehydrogenase-5 (LDH-5) overexpression in non-small-cell lung cancer tissues is linked to tumour hypoxia, angiogenic factor production and poor prognosis. British journal of cancer.

[46] Xiao Y, Motomura S, Podack ER (2008). APRIL (TNFSF13) regulates collagen‐induced arthritis, IL‐17 production and Th2 response. European journal of immunology.

[47] Hahne M (1998). APRIL, a new ligand of the tumor necrosis factor family, stimulates tumor cell growth. The Journal of experimental medicine.

[48] Vogt D, Gray C, Young W, Orellana S, Malouf A (2007). ARHGAP4 is a novel RhoGAP that mediates inhibition of cell motility and axon outgrowth. Molecular and Cellular Neuroscience.

[49] Hu, J., Meng, Y., Yu, T., Hu, L. & Mao, M. Ubiquitin E3 Ligase MARCH7 promotes ovarian tumor growth. *Oncotarget* (2015). 10.18632/oncotarget.3650 PMC4494930 25895127

[50] Bedard KM, Walter BL, Semler BL (2004). Multimerization of poly (rC) binding protein 2 is required for translation initiation mediated by a viral IRES. Rna.

[51] Bedard KM, Daijogo S, Semler BL (2007). A nucleo‐cytoplasmic SR protein functions in viral IRES‐mediated translation initiation. The EMBO journal.

[52] Nandal A (2011). Activation of the HIF prolyl hydroxylase by the iron chaperones PCBP1 and PCBP2. Cell metabolism.

[53] Foussard H (2010). LRCH proteins: a novel family of cytoskeletal regulators. PloS one.

[54] Zervos AS, Gyuris J, Brent R (1993). Mxi1, a protein that specifically interacts with Max to bind Myc-Max recognition sites. Cell.

[55] Jiang W (2015). Downregulation of VGLL4 in the progression of esophageal squamous cell carcinoma. Tumor Biology.

[56] Li H (2015). VGLL4 inhibits EMT in part through suppressing Wnt/β-catenin signaling pathway in gastric cancer. Medical Oncology.

[57] Jiao S (2014). A peptide mimicking VGLL4 function acts as a YAP antagonist therapy against gastric cancer. Cancer cell.

[58] Haase H (2005). Ahnak is critical for cardiac Ca (v) 1.2 calcium channel function and its β-adrenergic regulation. The FASEB Journal.

[59] Benaud C (2004). AHNAK interaction with the annexin 2/S100A10 complex regulates cell membrane cytoarchitecture. The Journal of cell biology.

[60] Davis T, Loos B, Engelbrecht A-M (2014). AHNAK: the giant jack of all trades. Cellular signalling.

[61] Shankar J (2010). Pseudopodial actin dynamics control epithelial-mesenchymal transition in metastatic cancer cells. Cancer research.

[62] de Morrée A (2012). Self-regulated alternative splicing at the AHNAK locus. The FASEB Journal.

[63] Xu Z (2005). Liver-specific inactivation of the Nrf1 gene in adult mouse leads to nonalcoholic steatohepatitis and hepatic neoplasia. Proceedings of the National Academy of Sciences of the United States of America.

[64] Wang W, Chan JY (2006). Nrf1 is targeted to the endoplasmic reticulum membrane by an N-terminal transmembrane domain Inhibition of nuclear translocation and transacting function. Journal of Biological Chemistry.

[65] Oh DH, Rigas D, Cho A, Chan JY (2012). Deficiency in the nuclear‐related factor erythroid 2 transcription factor (Nrf1) leads to genetic instability. FEBS Journal.

[66] Boado RJ, Tsukamoto H, Pardridge WM (1996). Evidence for Translational Control Elements Within the 5′-Untranslated Region of GLUT1 Glucose Transporter mRNA. Journal of neurochemistry.

[67] Zou Z, Eibl C, Koop H-U (2003). The stem-loop region of the tobacco psbA 5′ UTR is an important determinant of mRNA stability and translation efficiency. Molecular Genetics and Genomics.

[68] David CJ, Manley JL (2010). Alternative pre-mRNA splicing regulation in cancer: pathways and programs unhinged. Genes & development.

[69] Zhang J, Manley JL (2013). Misregulation of pre-mRNA alternative splicing in cancer. Cancer discovery.

[70] Mortazavi A, Williams BA, McCue K, Schaeffer L, Wold B (2008). Mapping and quantifying mammalian transcriptomes by RNA-Seq. Nature methods.

[71] Sugnet, C. W., Kent, W. J., Ares, M. & Haussler, D. In *Pacific Symposium on Biocomputing*.66-77 (World Scientific). 10.1142/9789812704856_0007 14992493

[72] Sakabe NJ, de Souza SJ (2007). Sequence features responsible for intron retention in human. BMC genomics.

[73] Ebihara K (1996). Intron retention generates a novel isoform of the murine vitamin D receptor that acts in a dominant negative way on the vitamin D signaling pathway. Molecular and cellular biology.

[74] Forrest ST, Barringhaus KG, Perlegas D, Hammarskjold M-L, McNamara CA (2004). Intron retention generates a novel Id3 isoform that inhibits vascular lesion formation. Journal of Biological Chemistry.

[75] Dytrych L, Sherman DL, Gillespie CS, Brophy PJ (1998). Two PDZ domain proteins encoded by the murine periaxin gene are the result of alternative intron retention and are differentially targeted in Schwann cells. Journal of Biological Chemistry.

[76] Schödel J (2011). High-resolution genome-wide mapping of HIF-binding sites by ChIP-seq. Blood.

[77] Mole DR (2009). Genome-wide association of hypoxia-inducible factor (HIF)-1α and HIF-2α DNA binding with expression profiling of hypoxia-inducible transcripts. Journal of biological chemistry.

[78] Bristow RG, Hill RP (2008). Hypoxia and metabolism: hypoxia, DNA repair and genetic instability. Nature Reviews Cancer.

[79] Schneider CA, Rasband WS, Eliceiri KW (2012). NIH Image to ImageJ: 25 years of image analysis. Nat Methods.

[80] Dobin A (2013). STAR: ultrafast universal RNA-seq aligner. Bioinformatics.

[81] Madan V (2015). Aberrant splicing of U12-type introns is the hallmark of ZRSR2 mutant myelodysplastic syndrome. Nature communications.

[82] Eisen MB, Spellman PT, Brown PO, Botstein D (1998). Cluster analysis and display of genome-wide expression patterns. Proc Natl Acad Sci USA.

