# Supplementary material for: Hypoxia is a Key Driver of Alternative Splicing in Human Breast Cancer Cells
Source: Sci Rep. 2017 Jun 22;7:4108. doi: 10.1038/s41598-017-04333-0 (PMC5481333; doi:10.1038/s41598-017-04333-0)
Supplement: Supplementary file 1 — Supplementary Information [file 41598_2017_4333_MOESM1_ESM.pdf]

Hypoxia is a Key Driver of Alternative Splicing in Human Breast Cancer Cells

Jian Han[1], Jia Li[1], Jolene Caifeng Ho[1], Grace Sushin Chia[1], Hiroyuki Kato[1], Sudhakar Jha[1], Henry Yang[1], Lorenz Poellinger[1,2] and Kian Leong Lee[1]

[1] Cancer Science Institute of Singapore, National University of Singapore, 117599, Singapore

[2] Department of Cell and Molecular Biology, Karolinska Institutet, Stockholm, SE-171 77, Sweden

Short title: Hypoxia Drives Alternative Splicing in Breast Cancer Cells

Supporting Information

Supplementary Figure S1. Classification and quantification of alternative splicing events. Schemas show types of splicing for **(a)** intron retention, **(b)** exon skipping, **(c)** 5’ splice sites, **(d)** 3’ splice sites and **(e)** alternative first exons. Reads corresponding to normal splicing are shown in green while specific alternative splicing events are shown in red. General gene structure is shown in blue with thick bars representing exons and thin lines indicating introns. Equations for the calculation of the splicing index (SI) for each splicing type are as indicated.

Supplementary Figure S2. Apoptosis assay and validation of RNA-Seq splicing events by qRT-PCR or RT-PCR. **(a)** Apoptosis analysis with Annexin V (Alexa Fluor 647) and SYTOX Blue staining showing the distribution of live, early and late apoptotic MCF7 cells in normoxia, 4h acute hypoxia and 24 hours chronic hypoxia. The x-axis shows fluorescence intensity for Annexin V (Alexa Fluor 647) dye, indicative of cells undergoing apoptosis, while the y-axis shows fluorescence intensity for SYTOX blue dye, indicative of dead cells both on logarithmic scales**.** Graph indicates the percentage of live (blue), early (orange) and late apoptotic (gray) cells out of the total number of cells for each treatment. Error bars indicate SEM for $n \geq 3$ replicates. **(b)** The expression of the alternative first exon isoforms for *MXI1* was measured using qRT-PCR and normalized against *PPIA* expression as the housekeeping reference control. All qRT-PCR analyses were performed in three biological replicates (n=3). Error bars indicate SEM while statistical significance was evaluated using Student's t-test. * indicates p-value<0.05, ** p-value<0.01. **(c)** Table shows target genes used for the comparison of ΔSI between RNA-Seq and that of qRT-PCR or RT-PCR. The ΔSI used is expressed as a percentage according to the formulas described in Supplementary Fig. S1 for each splicing type. **(d)** Scatter plot shows correlation between the splicing index determined by RNA-Seq analysis (x-axis) and that of qRT-PCR or RT-PCR (y-axis) for target genes spliced during hypoxia. R^2 indicates the coefficient of determination that quantifies how well the regression line fits the ΔSI data points with a perfect fit at 1.

Supplementary Figure S3. 5' and 3' RACE-PCR analysis of AHNAK isoforms in hypoxia. Agarose gel images show **(a)** 5' RACE-PCR and **(b)** 3'RACE PCR products of *AHNAK* amplified from MCF7 cells subjected to 24h hypoxia. Red arrows show two alternative first exon isoforms of AHNAK amplified by 5' RACE-PCR as indicated. 3'RACE-PCR gel products show predominantly one AHNAK isoform with little alternative splicing at the 3' end. **(c)** Schema of the gene structures of the canonical AHNAK-001 isoform and the novel alternative first exon AHNAK non-coding transcript. Thin black lines denote introns, beige boxes show non-coding exons and blue boxes indicate protein coding exons.

Supplementary Figure S4. Specificity of RT-PCR detection of isoform expression in normoxia/hypoxia and analysis of nonsense mediated decay (NMD) of isoforms. Gel images show RT-PCR of hypoxia treated MCF7 mRNA samples with (RT) and without (NO RT) reverse transcription of **(a)** *LDHA*, **(b)** *TNFSF13* and **(c)** *ARHGAP4*. N indicates normoxia, H4 hypoxia 4h, H24 hypoxia 24h and NTC denotes the no template control. Three biological replicates (n=3) are shown. Low molecular weight bands in NO RT reactions of *TNFSF13* correspond to primer dimers. **(d)** Gel images show RT-PCR of *EEF1G*, *PTGS2*, intron retentions (IR) of *LDHA*, *TNFSF13*, *ARHGAP4* and *TRMU* of MCF7 cells treated with 10ug/ml cycloheximide (CHX) for 6h. The housekeeping gene *EEF1G* was used as a loading and negative NMD control. PTGS2 was used as a positive NMD control.

Supplementary Figure S5. Specificity test of exon-exon junction primers used in exon skipping analysis. **(a)** Gel images show RT-PCR of *MARCH7*, *PCBP2* and *EIF4H* full length and exon skipping isoforms with single PCR products for each isoform-specific primer pair. The sanger sequencing results of **(b)** *MARCH7* full length (FL) and exon 9 skipping (**Δ**E9), **(c)** *PCBP2* full length (FL) and exon 11 skipping (**Δ**E11) and **(d)** *EIF4H* full length (FL) and exon 5 skipping (**Δ**E5) PCR products TA cloned into pGEM T-easy vectors are shown. Primer sequences used in this study are highlighted in grey. Exons are indicated using different colours. Partial sequences of the pGEM T-easy vector backbone are shown in black.

Supplementary Figure S6. (a) qRT-PCR analysis of the effect of cell density on hypoxia-regulated alternative splicing of *LDHA, VGLL4, MARCH7* and *PCBP2* genes. MCF7 cells were seeded at a density of 0.15, 0.3, 0.6 and 1.2 million cells as indicated. Line charts show qRT-PCR quantification of the expression of *LDHA, VGLL4, MARCH7* and *PCBP2* splicing isoforms over cell density in normoxia/hypoxia conditions after normalization to *PPIA* expression as the housekeeping reference control. **(b)** Line graph shows qRT-PCR quantification of *CA9* expression normalized to the *PPIA* housekeeping control in normoxia, 4h acute hypoxia and 24h chronic hypoxia at different cell densities as indicated. All qRT-PCR analyses were performed in three biological replicates (n=3). Error bars indicate SEM.

Supplementary Figure S7. (a) Full-length western blot picture of hypoxia-regulated RBPs used in Fig 1f. **(b)** Full-length gel picture of *LHDA* RT-PCR used in Fig 4a. **(c)** Full-length gel picture of *TNFSF13* RT-PCR used in Fig 4b. **(d)** Full-length gel picture of *ARHGAP4* RT-PCR used in Fig 4c. **(e)** Full-length gel picture of *LRCH3* RT-PCR used in Fig 6c. N indicates normoxia, H4 indicates hypoxia 4h, H24 indicates hypoxia 24h.

Supplementary Table S1. Global transcriptome alternative splicing events regulated during acute and chronic hypoxia. Table shows statistics of the number of alternative splicing events as determined by RNA-Seq analysis to be dysregulated in MCF7 cells during acute (4h) and chronic (24h) hypoxia compared to the normoxia control. Splicing types are as indicated and considered significant for a FDR of <0.01 and $|\Delta SI|$ of $\geq 15\%$.

Supplementary Table S2. Mapping summary of RNA-Seq reads obtained from hypoxia and normoxia treated MCF7 cells. Table shows statistics of the sequencing depth obtained from RNA-Seq of MCF7 cells subjected to normoxia, acute and chronic hypoxia. Over 90% of reads map uniquely to the human genome reference hg19 in all samples.

Supplementary Table S3. List of qRT-PCR and RT-PCR primers used for the quantification of isoform abundance. Forward and reverse primers were designed using NCBI Primer-BLAST and used for qRT-PCR and RT-PCR respectively as indicated.

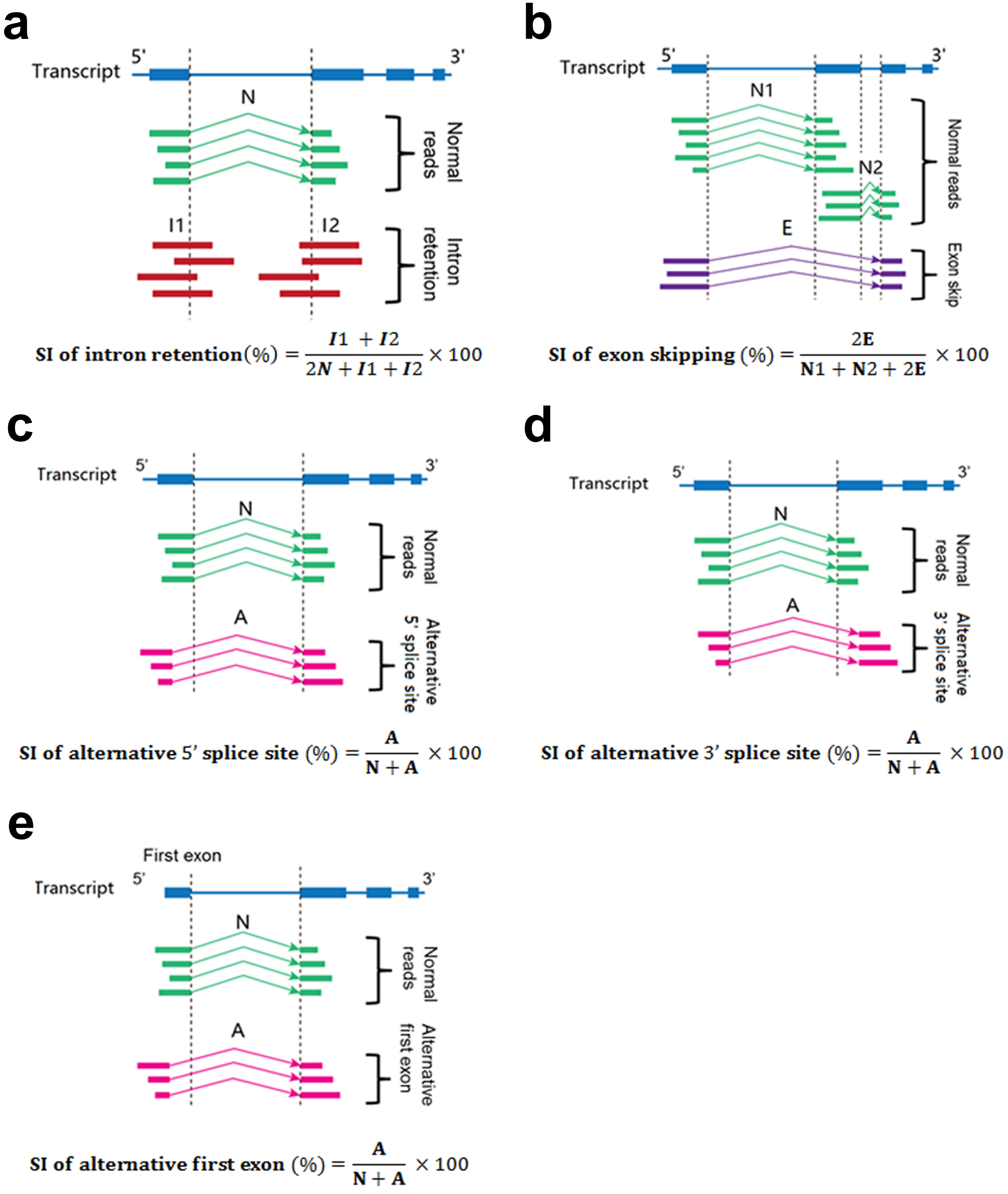

Supplementary Figure S1

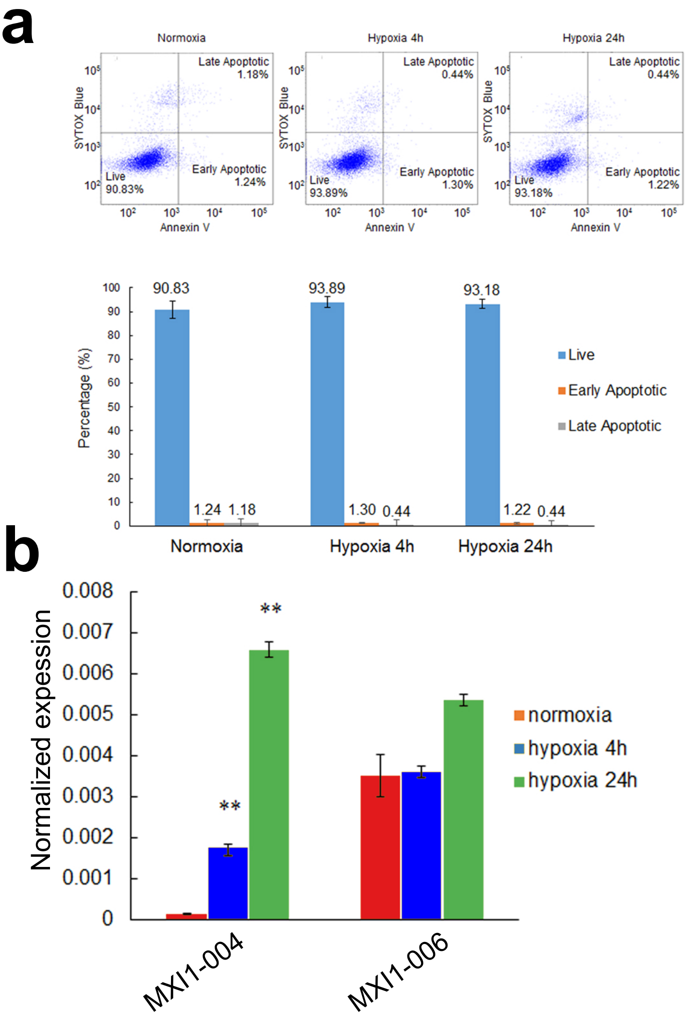

c

Gene	PCR	RNA-Seq
PCBP2	-8.82%	-15.98%
PFKFB3	-28.85%	-27.03%
VGLL4	32.52%	19.44%
EIF4H	-7.10%	-17.95%
MARCH7	13.58%	23.69%
FUT11	38.73%	47.48%
LDHA	54.29%	61.40%
NFE2L1	10.50%	15.77%
MXI1	-51.19%	-30.44%
AHNAK	68.10%	68.05%
TRMU	-9.15%	-28.24%
LRCH3	18.65%	25.78%
TNFSF13	30.25%	44.55%
ARHGAP4	20.44%	33.48%

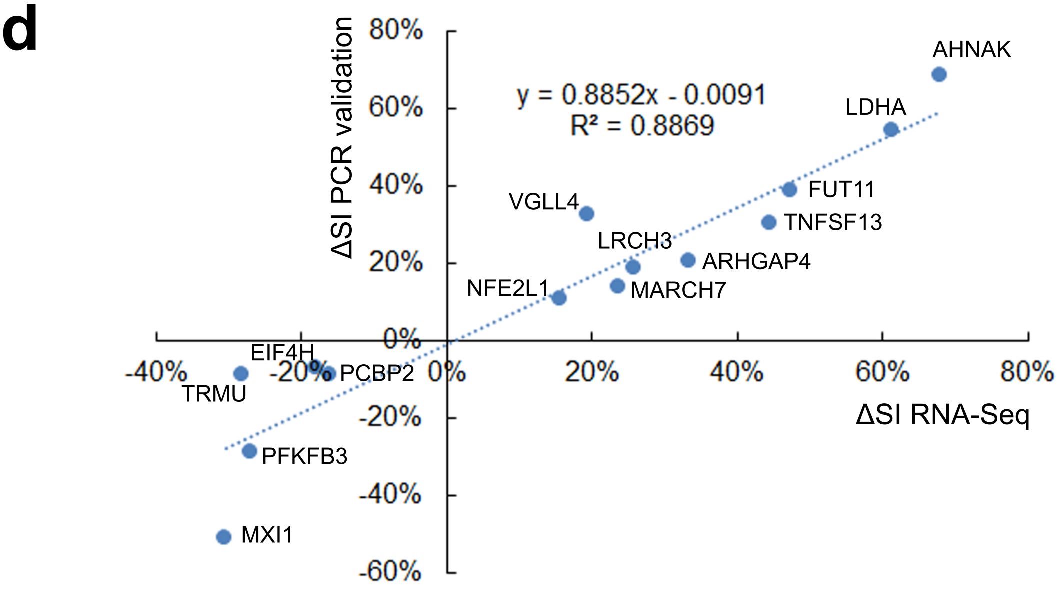

Supplementary Figure S2

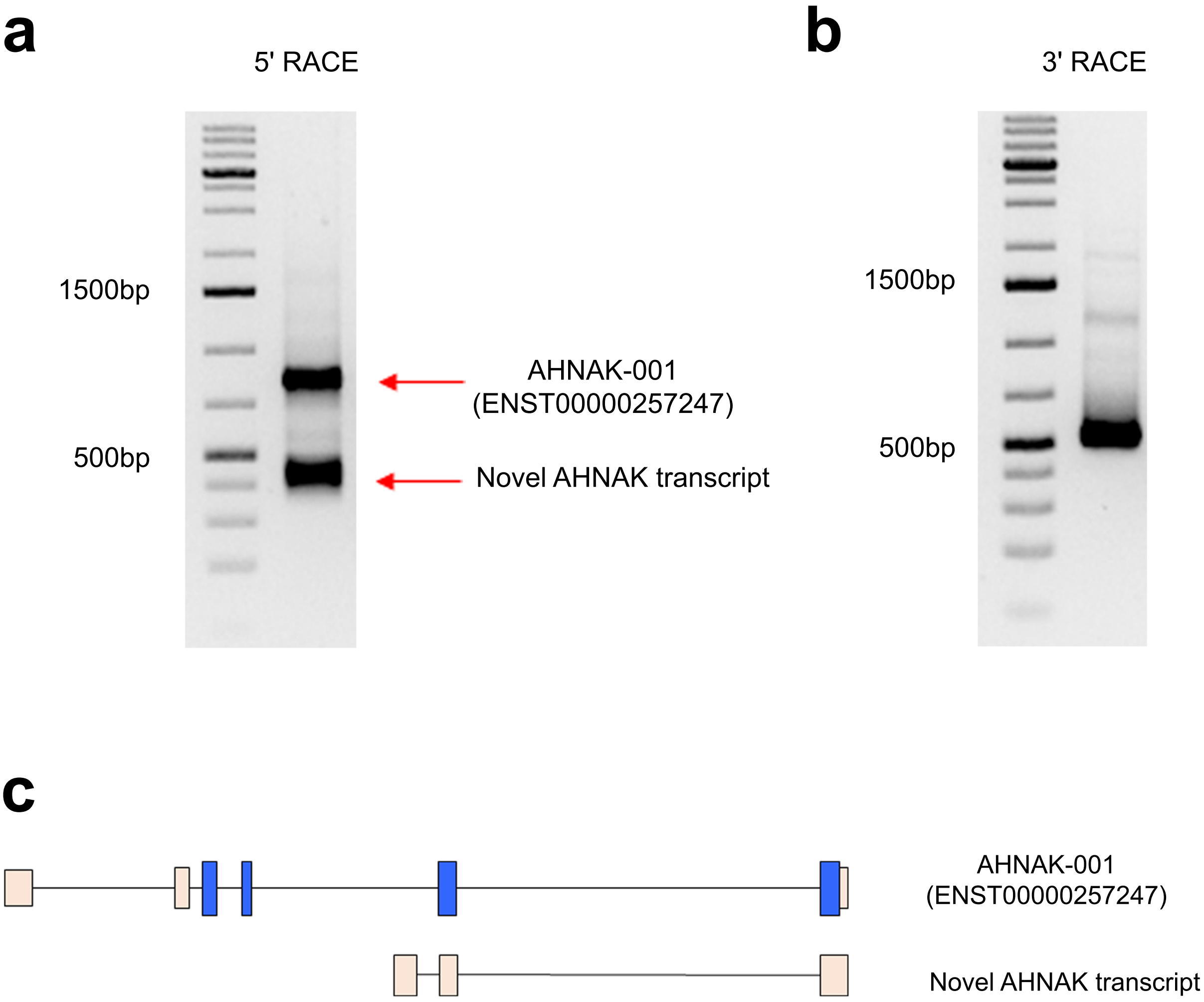

Supplementary Figure S3

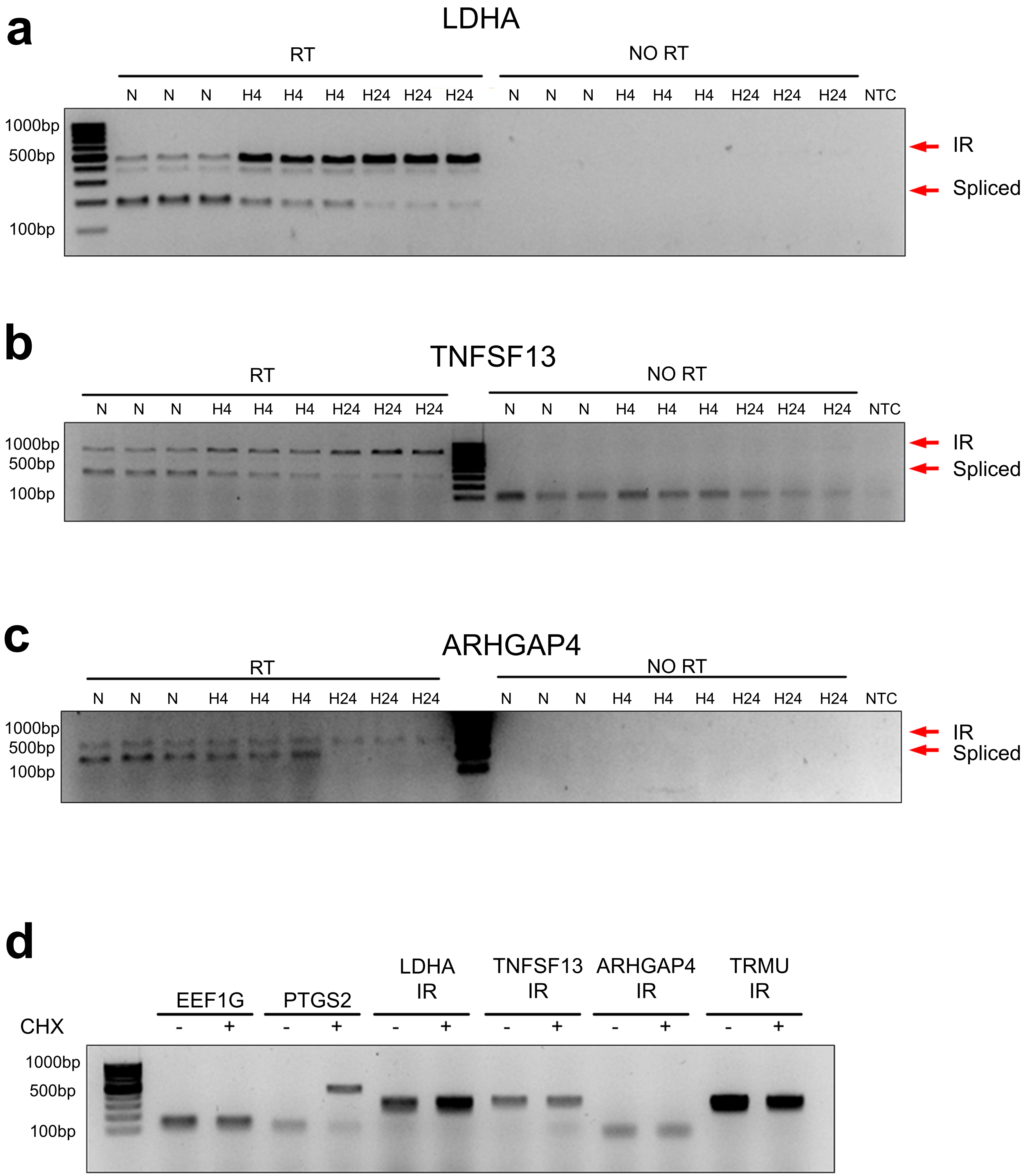

Supplementary Figure S4

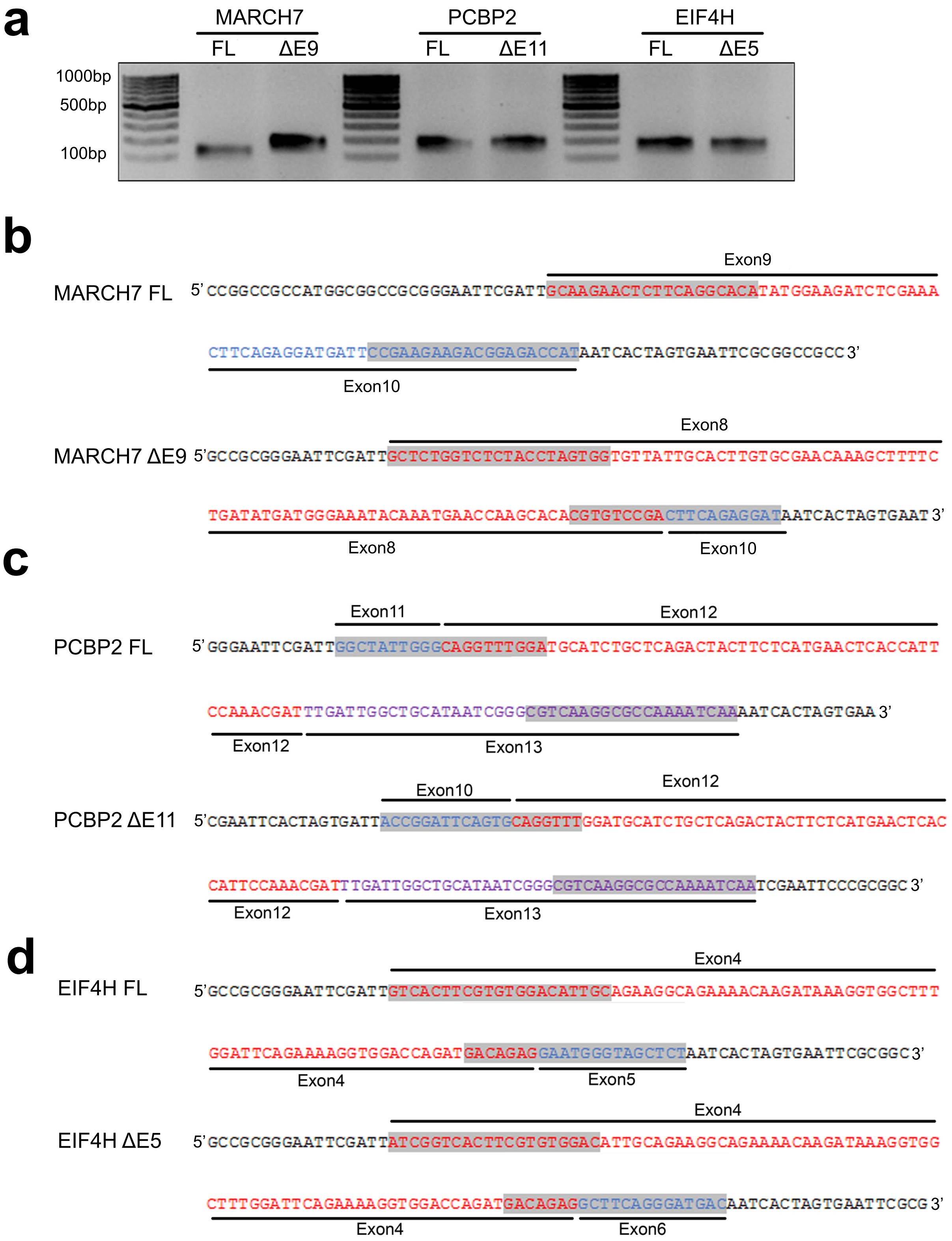

Supplementary Figure S5

a

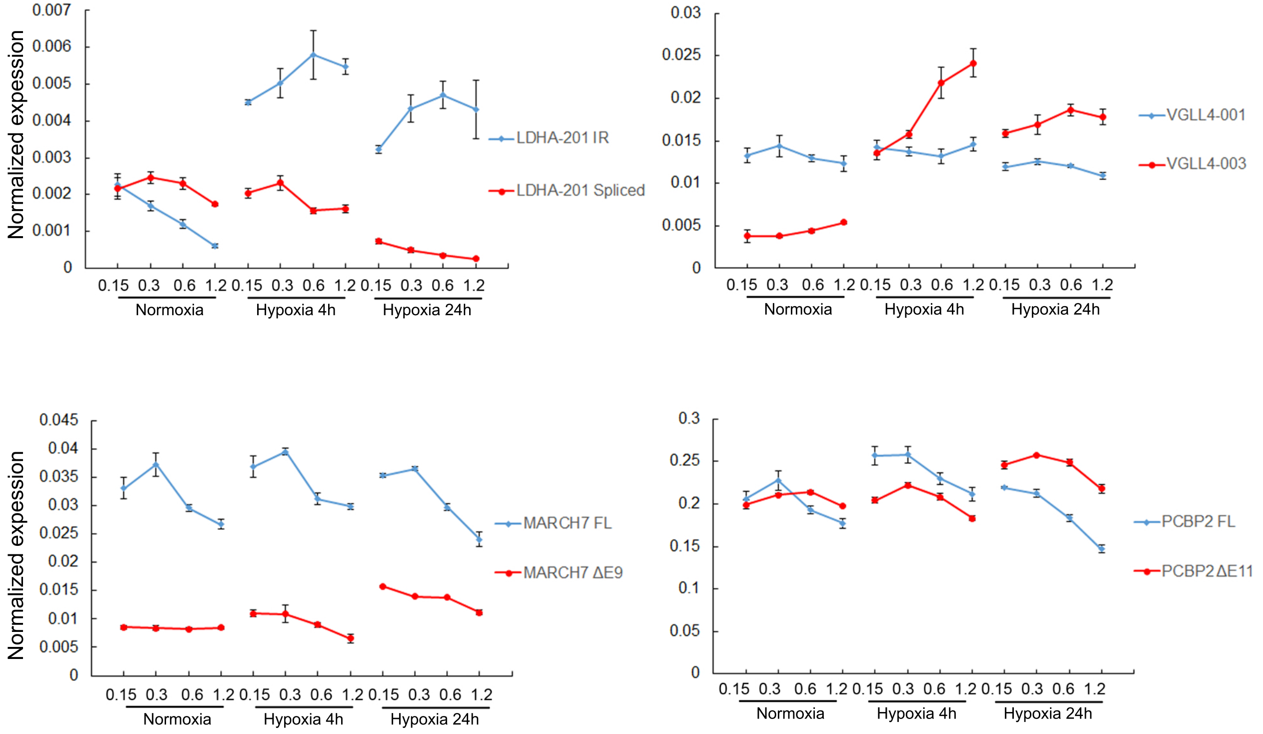

b

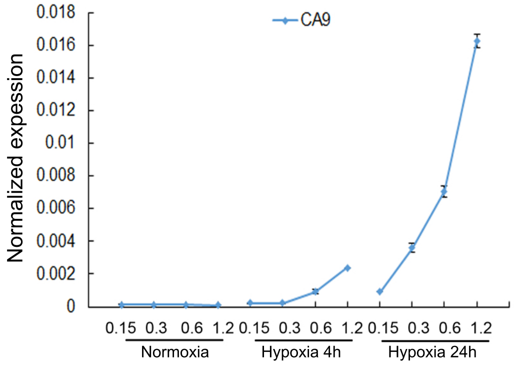

Supplementary Figure S6

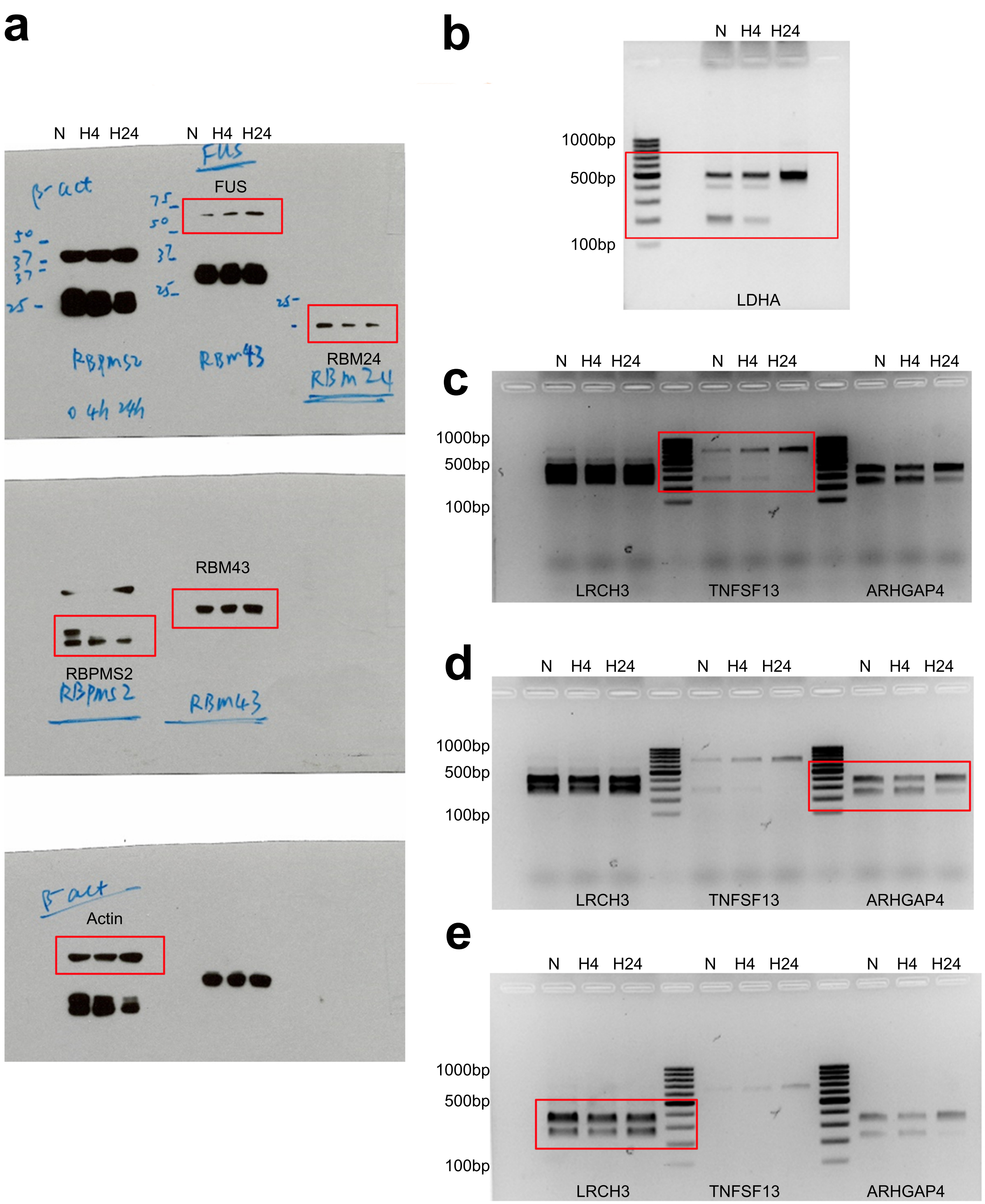

Supplementary Figure S7

Event Type	Hypoxia 4h vs Normoxia $\|\Delta SI\| \geq 15\%$ FDR<0.01			Hypoxia 24h vs Normoxia $\|\Delta SI\| \geq 15\%$ FDR<0.01		
	Novel	Known	Total	Novel	Known	Total
Exon skipping	77	170	247	71	188	259
Intron retention	1224	9	1233	1036	6	1042
Alternative 5' splice site	114	92	206	39	86	125
Alternative 3' splice site	145	109	254	117	89	206
Alternative first exon	22	43	65	11	41	52
Total	1582	423	2005	1274	410	1684

Supplementary Table S1

Samples	Total reads	Uniquely mapped reads	Uniquely mapped reads percentage
Normoxia	174,512,164	162,759,662	93.27%
Hypoxia 4h	176,070,129	165,128,413	93.79%
Hypoxia 24h	193,852,715	181,465,538	93.61%

Supplementary Table S2

Gene	Transcript and Splicing	Position	Forward(F)/ Reverse (R)	Sequence	Size
CA9	CA9-001	Exon5	F	CCTTTGCCAGAGTTGACGAG	109
CA9	CA9-001	Exon6	R	GACAGCAACTGCTCATAGGC	
RBM43	RBM43-001	Exon2	F	ATCCGACAAGAACCAAGGGA	127
RBM43	RBM43-001	Exon3	R	AGAGAGACTGTGAGTTCAGCAT	
FUS	FUS-001	Exon1	F	GGTACTCAGCGGTGTTGGAA	147
FUS	FUS-001	Exon3	R	ACTGCTCTGCTGGGAATAGC	
RBM24	RBM24-001	Exon2	F	CATTGATGGCAGAAAGGCCAA	105
RBM24	RBM24-001	Exon3	R	AGGGCTGGATGAAGTTGTTGA	
RBPMS2	RBPMS2-001	Exon6	F	CCCCTACCCTTTGTACACCAC	151
RBPMS2	RBPMS2-001	Exon7	R	ACGGTACTTCCATCCTTGCT	
LDHA	LDHA-201 Spliced & Intron Retention	Exon 1	F	CACCCAAACGTCGATATTCCT	148/356
LDHA	LDHA-201 Spliced & Intron Retention	Exon 2	R	AGCACCAACCCCAACAACTG	
LDHA	LDHA-201 Spliced & Intron Retention (used in Fig7c & Supplementary Fig S4d)	Exon 1	F	TGGCTGTGTCCTTGCTGTAG	
LDHA	LDHA-201 Spliced	Exon 1 & 2	R	ACCAAAAGGAATCTTAGCGTGG	163
LDHA	LDHA-201 Intron Retention (used in Fig7c & Supplementary Fig S4d)	Intron 1	R	TCTGTGAAGAGTGAAGGCCC	176
LDHA	LDHA-001	Exon 1	F	CGGATCTCATTGCCACGC	96
LDHA	LDHA-001	Exon 2	R	AGCTGATCCTTTAGAGTTGCCA	
LRCH3	LRCH3-004 Exon Skipping & Inclusion	Exon 14	F	CCCATCCAGAAGGTCTCAGC	200/272
LRCH3	LRCH3-004 Exon Skipping & Inclusion	Exon 17	R	TGTTGGTTTCTGCCCTGACA	
TNFSF13	TNFSF13-001 Spliced & Intron Retention	Exon 1	F	TTGCCCTCTGGTTGAGTTGG	219/545
TNFSF13	TNFSF13-001 Spliced & Intron Retention	Exon 2	R	TCCGGGATCTCTCCCCATTC	
TNFSF13	TNFSF13-001 Intron Retention (used in Supplementary Fig S4d)	Exon1	F	CAGTTGCCCTCTGGTTGAGT	210
TNFSF13	TNFSF13-001 Intron Retention (used in Supplementary Fig S4d)	Intron1	R	ATCCTCTCCCAGACACCCTC	
ARHGAP4	ARHGAP4-001 Spliced & Intron Retention	Exon 16	F	CTGAAGCTCTACTTCCGGAGC	172/260
ARHGAP4	ARHGAP4-001 Spliced & Intron Retention	Exon 17	R	AGGTGAAGAGGTAGCGCAGA	
ARHGAP4	ARHGAP4-001 Intron Retention (used in Supplementary Fig S4d)	Intron16	F	GGGTAGGGCACTTGCTGG	155
ARHGAP4	ARHGAP4-001 Intron Retention (used in Supplementary Fig S4d)	Exon17	R	GAAGAGGTAGCGCAGAACCA	
MARCH7	MARCH7-001 Exon Inclusion	Exon 9	F	GCAAGAACTCTTCAGGCACA	72
MARCH7	MARCH7-001 Exon Inclusion	Exon 10	R	ATGGTCTCCGTCTTCTTCGG	
MARCH7	MARCH7-001 Exon Skipping	Exon 8	F	GCTCTGGTCTCTACCTAGTGG	106
MARCH7	MARCH7-001 Exon Skipping	Exon 8&10	R	ATCCTCTGAAGTCGGACACG	
FUT11	FUT11-001 Intron Retention	Intron 1	F	GCACCCTCATGACAGCCTTA	70
FUT11	FUT11-001 Intron Retention	Exon 2	R	TCCCGATTCTGCAGGCATTT	
FUT11	FUT11-001 Spliced	Exon 1 & 2	F	CCACATCCCGGTAGACTCC	123
FUT11	FUT11-001 Spliced	Exon 2	R	TAGCGGGACAAGAAAGCCAA	
AHNAK	AHNAK Novel	Exon 1A	F	CTACGGTCCTCGTTTTGTGAGT	146
AHNAK	AHNAK Novel	Exon 2A	R	TGCTGGCTTCCTTCTGTTTGT	
AHNAK	AHNAK-005	Exon 5	F	ACGGGAAGCTGAAATTCGGT	88
AHNAK	AHNAK-005	Exon 5	R	CCTGTCTCATCATCGCTCCC	
AHNAK	AHNAK-001	Exon 4	F	AGACCTGGACCCGTGAAGT	141
AHNAK	AHNAK-001	Exon 5	R	GGGTGGAGACTGAAACTGCC	
MXI1	MXI1-004	Exon 0A	F	ACTCGATGGAGAAGCACATCA	123
MXI1	MXI1-006	Exon 1B	F	CATGGAGCGGGTGAAGATGA	101
MXI1	MXI1-006 & MXI1-004	Exon 2	R	ATGAAGAGGCGTAGCCATGT	
NFE2L1	NFE2L1-006	Exon 1A	F	GGGAAGTAGCACTTGTTCGCT	156
NFE2L1	NFE2L1-001	Exon 1B	F	GACGCCGAGCTAAGCAGTT	81
NFE2L1	NFE2L1-001 & 006	Exon 2A	R	GCCTTTCGTCTTGGGTCAGA	
TRMU	TRMU-001 Spliced & Intron Retention (used in Fig S4d)	Exon 2	F	GGTCTGTACTGCCGACAAAGA	
TRMU	TRMU-001 Spliced	Exon 3	R	CAGGATTGGGAGTCCTTCCTTT	155
TRMU	TRMU-001 Intron Retention (used in Fig S4d)	Intron 2	R	GGCTGGTTACCGGATCCTTC	187

PCBP2	PCBP2-202 Exon Inclusion	Exon 11 & 12	F	GGCTATTGGGCAGGTTTGGA	107
PCBP2	PCBP2-202 Exon Skipping	Exon 10 & 12	F	ACCGGATTCAGTGCAGGTTT	110
PCBP2	PCBP2-202 Exon Skipping & Inclusion	Exon 12	R	TTGATTTTGGCGCCTTGACG	
EIF4H	EIF4H-001 Exon Skipping	Exon 4A	F	ATCGGTCACTTCGTGTGGAC	101
EIF4H	EIF4H-001 Exon Skipping	Exon 4 & 6	R	GTCATCCCTGAAGCCTCTGTC	
EIF4H	EIF4H-001 Exon Inclusion	Exon 4B	F	GTCACTTCGTGTGGACATTGC	179
EIF4H	EIF4H-001 Exon Inclusion	Exon 4 & 5	R	AGAGCTACCCATTCCTCTGTC	
PFKFB3	PFKFB3-001	Exon 1 & 2	F	CCCTTCAGGAAAGCCTGTGG	139
PFKFB3	PFKFB3-002	Exon 1B	F	CCGAGTGCAGAAGATCTGGG	179
PFKFB3	PFKFB3-001 & 002	Exon 2B	R	CTTTTGTGGGGACGCCAATC	
VGLL4	VGLL4-001	Exon 2A	F	TGACGAAAAACGCGAAGCTG	150
VGLL4	VGLL4-001	Exon 3A	R	TCTAGGTCCTCGTCACCTGG	
VGLL4	VGLL4-003	Exon 1B	F	TTGGGGCAAAAGCAAAGAGC	180
VGLL4	VGLL4-003	Exon 2B	R	CGGCAGGGTCTGTATTCTGG	
PPIA	PPIA-001	Exon1	F	ACGGCGAGCCCTTGG	223
PPIA	PPIA-001	Exon2	R	TTTCTGCTGTCTTTGGGACCT	
EEF1G	EEF1G-001	Exon2	F	AACCGCACCCCTGAATTTC	68
EEF1G	EEF1G-001	Exon3	R	CCATCATCACCCTCAAATGCT	
PTGS2	PTGS2-001	Exon7	F	TGCCTGGTCTGATGATGTATGCCA	306
PTGS2	PTGS2-001	Exon8	R	TTTGAAAGGTGTCAGGCAGAAGGG	

Supplementary Table S3
